# Multistep Relaxation Pathway from Glass to Crystal Development in Poly(l‑lactide)

**DOI:** 10.1021/acs.macromol.5c02464

**Published:** 2025-12-12

**Authors:** Lorenzo Augusto Rocchi, Elisa Sturabotti, Andrea Martinelli, Daniele Cangialosi, Valerio Di Lisio

**Affiliations:** † Department of Chemistry, 9311 Sapienza University of Rome, P.le Aldo Moro 5, 00185 Rome, Italy; ‡ 226245 Donostia Internation Physics Center (DIPC), Paseo Manuel Lardizábal 4, 20018 Donostia-San Sebastián, Spain; § Centro de Física de Materiales, CSIC-UPV/EHU, Paseo Manuel de Lardizábal 5, 20018 Donostia-San Sebastián, Spain

## Abstract

The thermal transitions of amorphous poly­(l-lactide) (PLLA) were investigated by combining standard calorimetry (DSC), fast scanning calorimetry (FSC), and infrared spectroscopy. By following the enthalpic evolution, the glass transition temperature (*T*_g_) change, and the chains’ conformational behavior of PLLA during sub-*T*_g_ and supra-*T*_g_ annealing isotherms, a transition was identified in the supercooled liquid (SCL) phase—the so-called SCL transformation—occurring before crystallization. SCL transformation kinetics was assessed and introduced into the relaxation map of amorphous PLLA, revealing a hierarchical pathway. The SCL transformation allows the unstable PLLA-quenched liquid to relax toward a metastable SCL with a lower free energy. This occurs through conformational rearrangement of a small fraction of *gg* conformers into the more stable *gt* form. Ultimately, SCL kinetics was compared to PLLA liquid dynamics, providing a suggestive scenario where the SCL transformation is mediated by both α-relaxation and non-α relaxation mechanisms—the Slow Arrhenius Process (SAP).

## Graphical abstract

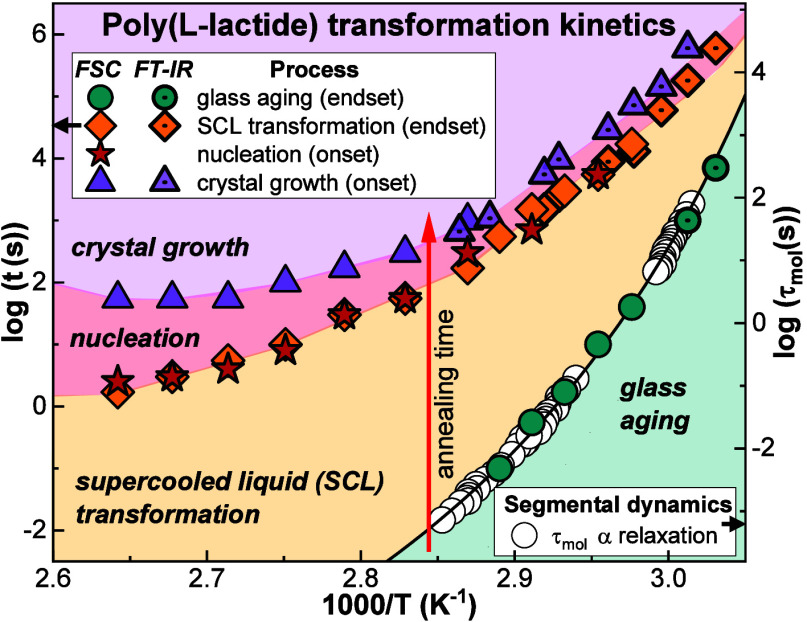

## Introduction

Poly­(l-lactide) (PLLA) is a biobased and biodegradable polyester obtained from renewable resources such as sugar cane, potato, and corn starch. Low price, good processability, and excellent mechanical properties have made PLLA one of the most produced bioplastics in recent years. In 2024, it accounted for 35.8% of global bioplastics production capacities, with projections expecting a rise to 40.6% by 2029.^1^ Due to its intriguing features, PLLA has emerged as the epitome of a “multipurpose material”, attracting much attention from both academic and industrial communities. Compared to other commercially available thermoplastic biopolymers, such as poly­(3-hydroxy butyrate) (PHB),^2^ poly­(butylene succinate) (PBS),^3^ or poly­(caprolactone) (PCL),^4^ PLLA exhibits the distinctive feature of exhibiting a glass transition temperature (*T*_g_) that is higher than, although close to, room temperature. Hence, depending on the processing conditions of the melt, the final fate of this material will be either to crystallize^5,6^ or, if the cooling rate is fast enough, to fully vitrify.^7,8^ Consequently, long-term performances of the final PLLA product, such as mechanical strength,^9,10^ optical clarity,^11^ barrier properties,^12,13^ biodegradability, and bioresorption,^14^ are significantly influenced by the relative content of crystal and amorphous phases^5,15,16^ and by all the nonequilibrium kinetic phenomena occurring in the glassy state.^17^

Over the last two decades, a significant area of study on crystallizable polymers has focused on how the thermodynamic state of the glass influences crystallization kinetics. Being a nonequilibrium state, the thermodynamic properties of a glass (such as density, enthalpy, and entropy) are not uniquely defined by temperature but spontaneously evolve over time toward the closest equilibrium state, i.e., the supercooled liquid (SCL). This kinetic process of structural reorganization is defined as physical aging,^18,19^ leading to glass densification and enthalpy loss. For PLLA, as well as for other crystallizable polymers such as poly­(ethylene terephthalate) (PET),^20–22^ it has been found that physical aging enhances crystallization kinetics, hypothesizing that chain rearrangements in the glassy state promote low-temperature homogeneous crystal nucleation, thereby enhancing crystallization on heating. This vision was challenged by Na et al.,^23^ who observed PLLA crystallization enhancement even after isothermal annealing above *T*_g_. This finding ruled out the possibility that enhancement of cold crystallization is exclusively induced by physical aging in the glassy state, suggesting instead that an additional relaxation process active in the supercooled liquid also affects nucleation kinetics. To further explore the underlying mechanisms behind polymer nucleation, even in the absence of glass densification, Androsch et al.^8,24–26^ conducted an extensive investigation into PLLA crystal nucleation at high cooling rates from the melt. In these studies, it was observed that the temperature dependence of the nucleation rate appears to be independent of whether the amorphous phase is in the glass or SCL state, although the exact nucleation mechanism remains unknown. Ultimately, Di Lisio et al.^27^ provided a microstructural insight into PLLA amorphous transformations by employing temperature-modulated FTIR spectroscopy (TM-FTIR).^28,29^ Analogously to temperature-modulated calorimetry (TMDSC),^30,31^ this technique enabled the observation of an unexpected nonreversing transformation occurring in between the glass transition and cold crystallization. This suggested that the SCL phase obtained by fast cooling undergoes conformational rearrangements toward a relaxed liquid with a higher concentration of low-energy conformers before crystallization.

In this work, the thermal and microstructural aspects associated with the conformational rearrangement of the PLLA liquid phase,^27^ referred to as the SCL transformation, are investigated through a combination of differential scanning calorimetry (DSC), fast scanning calorimetry (FSC), and time-resolved Fourier-transform infrared spectroscopy (TR-FTIR). While the effects of the SCL transformation on PLLA physical properties are known but rarely reported,^23,32^ its fundamental thermodynamics and kinetics remain uncharacterized. Therefore, the objective of this study is to map SCL transformation kinetics, to establish its thermodynamic stability, and to determine its influence on the liquid’s segmental dynamics and ultimately on nucleation and crystallization kinetics.^24,28,33^

A comprehensive map of the overall transformation kinetics of amorphous PLLA was built, implementing calorimetric and infrared data with liquid dynamics assessed by broadband dielectric spectroscopy from Wang and co-workers.^33^ A suggestive scenario emerges, where a secondary molecular mechanism, addressed as the Slow Arrhenius Process (SAP),^34^ appears to be the key to understanding the temperature dependence and time scales of the SCL transformation.

Ultimately, these findings challenge the conventional model of liquid relaxation, where the main structural relaxation (α-relaxation) has been considered to be the unique mechanism driving thermodynamically unstable systems to equilibrium. We believe that the introduction of additional relaxation mechanisms could improve the understanding of the preordering steps that supercooled liquids undergo during polymer crystallization.

## Experimental Part

### Materials

Poly­(l-lactide) (PLLA) (Purasorb PL32, *M*_w_ = 521,000 g mol^–1^, η_inh_ = 3.27 dL g^–1^ in CHCl_3_ 0.1 g dl^–1^ at 25 °C, and [α]_D_^25^ = −157.6° in CHCl_3_ 0.1 g dl^–1^ at 20 °C) and poly­(D, l-lactide) (PDLLA) (Purasorb PDL45) were kindly supplied by Corbion Purac (Gorinchem, The Netherlands). CHCl_3_, spectroscopy-grade KBr, biphenyl, and benzoic acid were purchased from Sigma-Aldrich (St. Louis, Missouri) and used as received.

### Differential and Fast Scanning Calorimetry

Differential scanning calorimetry (DSC) was performed using two different calorimeters, specifically a TA Instruments DSC Q2000 and a Mettler Toledo DSC 822e. Both DSC instruments were operated under similar conditions to ensure the consistency of the experimental results. The sample chambers were purged with dry nitrogen at a constant flow rate of 50 mL min^–1^ throughout the experiments. The complete calibration protocol for temperature, heat flow, and thermal lag was performed using indium and zinc. Specimens of about 10 mg were sealed in 40 μL aluminum pans.

Fast scanning calorimetry (FSC) was carried out employing a Mettler–Toledo Flash Differential Scanning Calorimeter (Flash DSC 1) complemented by a Huber TC100 intracooler. Dry nitrogen was introduced into the sample chamber at a constant flow rate of 20 mL min^–1^. Sample specimens were prepared by placing an approximately 100 ng of polymer piece with a single-haired brush directly onto a Mettler–Toledo UFS 1 chip. The sample mass, necessary for calculating the specific heat capacity curves, has been indirectly determined from FSC heat flow curves by using eq 1:

$$m=\frac{\Delta HF(T_{g})}{q\Delta c_{p}(T_{g})}$$ 1

where Δ*HF*(*T*_g_) is the heat flow step at the glass transition of a generic heating scan, *q* is the heating rate, and Δ*c*_p_(*T*_g_) is the tabulated specific heat capacity difference between the liquid and glass states at the same temperature.^35^ Chip temperature calibration was performed using the melting point of indium at a heating rate of 1000 K s^–1^. The thermal lag was determined to be +5.5 K for the chip where PLLA specimens were placed and +2.0 K for the chip used for PDLLA. Both *T*_a_ and temperature scales of FSC heating and cooling scans were corrected according to the previous values.

Calorimetric measurements comprised four different thermal protocols, that are, isothermal annealing, asymmetry of approach, Tammann two-stage nuclei development, and step-response protocol. To eliminate the influence of thermal history, all DSC and FSC annealing experiments were initiated at 483 K, above the melting point of PLLA. To establish a reference scan for FSC, the samples were cooled at 1000 K s^–1^ from 483 to to 273 K and reheated to 483 K at the same rate. For the DSC reference curve, the same temperature interval was adopted, with heating/cooling rates of +10/–30 K min^–1^. Unless otherwise specified, a common cooling/heating rate of 1000 K s^–1^ was adopted for FSC scans, whereas a heating rate of 10 K min^–1^ and a cooling rate of −30 K min^–1^ were adopted for DSC scans. To check the thermal stability of samples, reference curves were recorded always before and after the end of the thermal protocols.

Isothermal annealing protocols have been performed on both FSC and DSC, comprising a cooling segment from 483 K to the annealing temperature, *T*_a_, an isotherm of a defined annealing time, *t*_a_, a cooling from *T*_a_ up to 273 K, and a final heating scan up to 483 K, defining the analysis scan. The protocol was repeated by varying the *T*_a_ and *t*_a_, depending on the measurement. Table 1 reports annealing temperatures and annealing time spans used for FSC and DSC. To enhance the detection of subtle thermal transitions and improve the signal-to-noise ratio, FSC experiments were replicated 5–10 times, depending on the specific time and temperature conditions. The final signals, along with their elaboration for Δ*H* determination, were obtained by curve averaging. A maximum Δ*H* error of ±10% was obtained.

**Table 1:** Annealing Temperatures *T*_a_ and Annealing Time *t*_a_ Intervals, Chosen for FSC, DSC, and FTIR

Technique	*T*_a_ (K)	*t*_a_ (s)
DSC, isothermal annealing	335.0–341.0 every 2 K	100–10^4^
FSC, isothermal annealing	336.2–346.2 every 2.5 K; 348.7–378.7 every 5 K	0.1–10^5^
FSC, Tammann two-stage	338.7–378.7 every 5 K	0.1–10^5^
FSC, up-jump	368.7	0.1–170
TR-FTIR, isothermal annealing	329.2–349.2	30–10^6^

The formation of PLLA homogeneous crystal nuclei was investigated by the two-stage Tammann protocol. The following technique, adopted only on FSC, is a modification of the isothermal annealing protocol, introducing a postannealing treatment before the analysis scan. After the first isothermal annealing at *T*_a_ for *t*_a_, defined as the nucleation stage, the sample was heated at 100 K s^–1^ up to the growth-stage temperature *T*_c_ = 398.7 K and kept for 100 s, where, according to classical nucleation theory, the nuclei develop into crystals. Finally, the sample was cooled at 273 K, and the thermal behavior was acquired with the analysis scan up to 483 K. *T*_a_ and *t*_a_ values employed in this method are summarized in Table 1.

To explore additional free-energy minima beyond those of the conventional supercooled liquid and the crystal, the present study adopted a modified version of the standard annealing protocol, referred to as the asymmetry of approach. This protocol assesses thermodynamic stability by subjecting samples to the same *T*_a_ values from various initial states. The asymmetry of approach, performed on FSC, comprised two different isothermal annealing at the same *T*_a_ = 368.7 K, defined as down-jump and up-jump. The down-jump has been carried out with the standard isothermal annealing protocol (approaching *T*_a_ from high temperature), whereas the up-jump included a preannealing treatment. Instead of reaching *T*_a_ from the melt, the sample was previously cooled at 353.7 K and held for 100 s. After the preannealing step, the sample was heated at *T*_a_ = 368.7 K and kept for different *t*_a_ (see Table 1), followed by cooling at 273 K and the final analysis scan up to 483 K.

By employing FSC and DSC step-response analyses, the spontaneous fluctuations related to the α-relaxation were assessed over a time span of about five decades. To accomplish this, five separate step-response experiments were carried out for each specimen. The step-response protocol consisted of a series of stimulation cycles of duration *t*_p_, each consisting of an initial up-jump of Δ*T* = 2 K at a heating rate *q*_H_, followed by an isotherm of duration *t*_iso_. The experimental parameters for the step-response protocols are detailed in Table S1. By analyzing the thermograms, the real, *c*_p_^′^, and imaginary, *c*_p_^″^, contributions, as well as the modulus, *c*_p_^rev^, of the complex susceptibility, *c*_p_^*^(ω), have been calculated for the base angular frequency ω_o_ = 2π*t*_p_^–1^ and higher-order harmonics ω = *k*ω_o_ (*k* = 1,2,3, ···) of each experiment and for each stimulation period *t*_p_ by the discrete fast Fourier-transform (DFFT) relationship, shown in eq 2:

$$c_{p}^{*}(\omega )=\frac{\sum_{t=0}^{t_{p}}HF(t)\cdot e^{i\omega t}\cdot \Delta t}{\sum_{t=0}^{t_{p}}q_{H}(t)\cdot e^{i\omega t}\cdot \Delta t}$$ 2

where HF­(*t*) is the instantaneous heat flow recorded during the step protocol. Finally, the dynamic glass transition temperature *T*_g_^dyn^ that gives information on the temperature dependency of the relaxation time τ­(*T*_g_^dyn^) = ω^–1^(*T*_g_^dyn^) was evaluated at the midpoint of *c*_p_^′^(ω).

### Fourier-Transform Infrared Spectroscopy

Polymer films suitable for FTIR analysis were prepared by spin-coating a PLLA solution in chloroform (25 mg mL^–1^) onto KBr disks using a PWM32 spin-coater (Headway Research) at 2000 rpm for 120 s. After drying under vacuum, the films exhibited a thickness of approximately 6–10 μm. To eliminate mechanical stresses induced by spin-coating, the films were melted at 453 K for 2 min and promptly quenched in liquid nitrogen. The same procedure was repeated for the PDLLA sample. The prepared specimens were mounted in a SPECAC heating cell controlled by a HELLMA thermo-programmer. To ensure the accuracy of the sample temperature, a calibration was conducted using the melting points of biphenyl (*T*_m_ = 344 K) and benzoic acid (*T*_m_ = 395 K), both dispersed in KBr disks. The calibration demonstrated a sample temperature accuracy of ±0.4 K, validating the reliability and accuracy of the experimental setup. Transmission infrared spectra were sequentially acquired by employing a Nicolet 6700 FTIR spectrometer. Spectra were collected over both time and temperature in the mid-infrared region (4000–400 cm^–1^) with a spectral resolution of 4 cm^–1^, and through coadding 40 scans per spectrum (sampling rate of 30 s per spectrum). After the quenching procedure, the sample was subjected to a heating ramp from room temperature at a constant rate of 1.5 K min^–1^ until it reached *T*_a_. The sample was then held at this temperature for a variable time interval *t*_a_ while spectra were recorded. The experimental parameters of the TR-FTIR protocols are reported in Table 1. Detailed sample information is reported in Table S2.

## Results and Discussion

Figure 1 shows DSC thermograms of amorphous PLLA, previously cooled at 30 K min^–1^ from 483 K and annealed in the supercooled liquid state at an annealing temperature *T*_a_ = 337 K for various times (Figure 1a), along with enthalpy variations of the main thermal events, shown in Figure 1b. Figure S1 of the SI reports additional DSC thermograms of quenched PLLA annealed at *T*_a_s of 335, 339, and 341 K. The thermograms (Figure 1a), recorded after isothermal annealing for various times, *t*_a_, reveal three main thermal events: the glass transition step (*T*_g_) at around 332 K, the exothermic cold crystallization (*T*_cc_) between 370 and 430 K, and the endothermic melting (*T*_m_) at approximately 453 K. Notably, the cold-crystallization peak is bimodal under all annealing conditions, comprising a shoulder at 380 K and a peak at 390–405 K. This is due to the well-known polymorphism of PLLA, which crystallizes in both the metastable α’ form and the stable α phase, depending on the crystallization temperature.^36^ As annealing proceeds, cold crystallization is favored, as suggested by a subtle shift of the main exothermic peak toward lower temperatures and by its increased intensity, while the onset remains nearly invariant. The hastening of cold-crystallization kinetics also involves a gradual increase of the total crystallization enthalpy Δ*H*_cc_, plotted in Figure 1b, from −37.5 J g^–1^ for a quenched sample to a constant value of −40.2 J g^–1^ for annealing isotherms longer than 7200 s. This was accompanied by a concurrent increase in melting enthalpy Δ*H*_m_ from 38.1 to 40.7 J g^–1^. The almost complete equivalence between −Δ*H*_cc_ and Δ*H*_m_ confirms that, before the heating scan, PLLA specimens were totally amorphous; thus, the used annealing parameters did not lead to crystallization.

**Figure 1:**
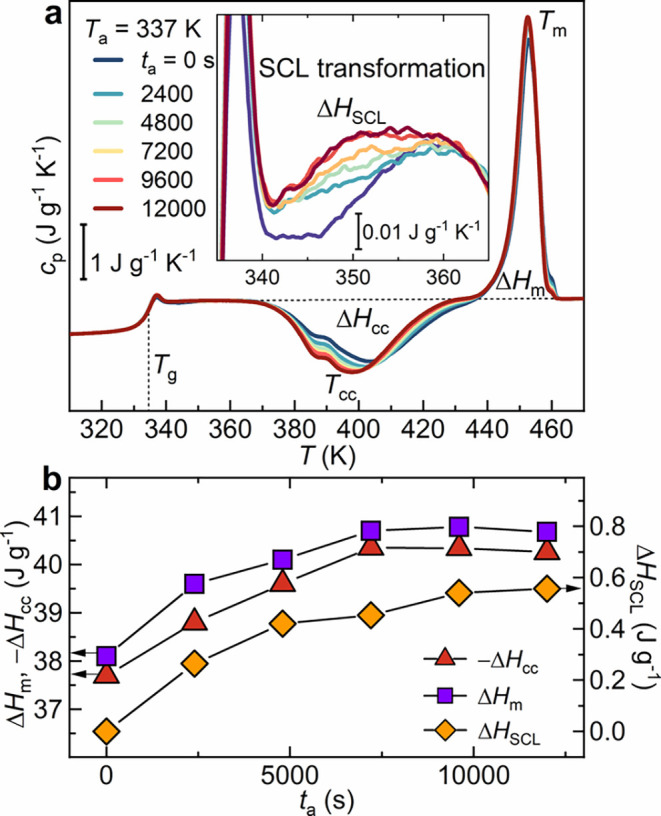
(a) DSC thermograms of amorphous PLLA annealed at 337 K for different times *t*_a_, showing glass transition steps, cold crystallization, and melting peaks. The inset highlights the SCL transformation. (b) Enthalpy of cold crystallization Δ*H*_cc_, melting Δ*H*_m_, and SCL transformation Δ*H*_SCL_ as a function of the annealing time.

A more meticulous examination of the temperature range between the glass transition and cold crystallization (see the inset of Figure 1a), at which PLLA exists in the supercooled liquid (SCL) state, reveals the presence of a broad and subtle endothermic transition. The latter, referred to henceforth as the “SCL transformation”, spans between 340 and 370 K for a DSC trace recorded at 10 K min^–1^. The time dependence of the enthalpy change caused by the SCL transformation over the annealing time, defined Δ*H*_SCL_ and illustrated in Figure 1b, appears to mimic that of Δ*H*_cc_ and Δ*H*_m_, stabilizing after 7200 s at a value of 0.57 J g^–1^.

Here, it is worth pointing out that an exact evaluation of the enthalpy involved in the SCL transformation remains elusive by standard DSC analysis due to its partial superimposition with the incipient cold crystallization occurring at slightly higher temperatures. For this reason, its assessment remains, to a certain extent, qualitative.

To obtain detailed quantitative information about the time-dependent behavior of amorphous PLLA, the separation of the SCL transformation from cold crystallization is required. To this aim, we resourced to fast scanning calorimetry (FSC), which allows heating and cooling rates on the order of thousands of K s^–1^, thereby enabling suppression of crystallization during heating scans.^37,38^ This helps reveal subtle thermal features occurring within the supercooled liquid region.

Figure 2a,c,e shows the FSC heating scans of quenched PLLA, annealed for different times (*t*_a_) at three annealing temperatures (*T*_a_). At the experimental heating rate of 1000 K s^–1^, the *T*_g_ of PLLA is about 343 K (considerably larger than 332 at 10 K min^–1^), taken at the midpoint of the *c*_p_ step. To highlight the thermal effects induced by annealing, the specific heat capacity difference (*c*_p_ – *c*_p_^ref^) between an annealed sample heating scan *c*_p_(*T*_a_,*t*_a_) and the reference scan *c*_p_^ref^(*T*_a_,0) (quenched sample at −1000 K s^–1^ and immediately reheated at the same rate) is presented in Figure 2b,d,f. Figure S2 of the SI plots the *c*_p_ – *c*_p_^ref^ curves recorded after annealing for all explored *T*_a_.

**Figure 2:**
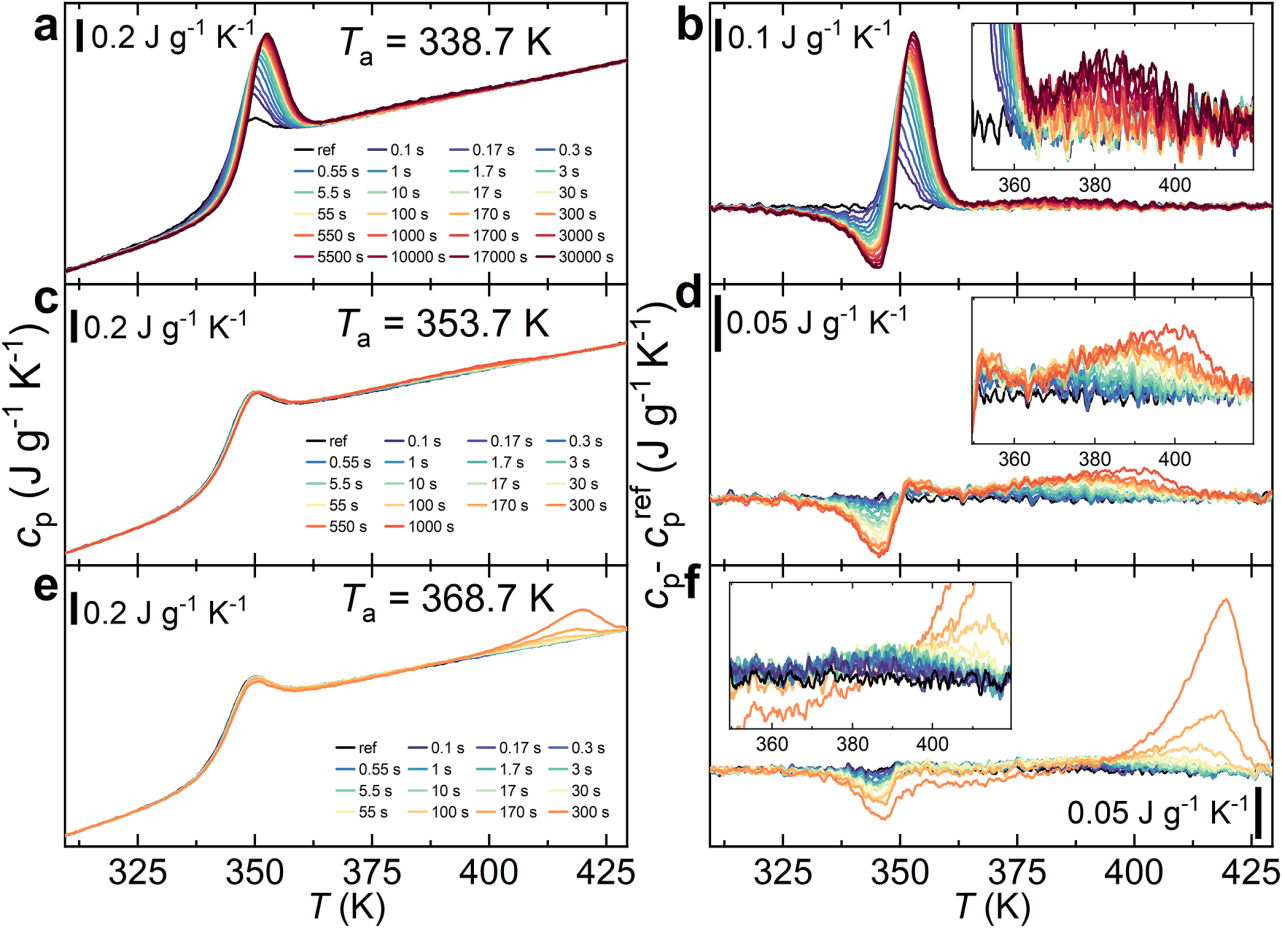
FSC heating scans (1000 K s^–1^) of quenched PLLA after annealing at different temperatures (*T*_a_) and times (*t*_a_). Panels (a–e) show the measured specific heat capacity (*c*_p_). Panels (b, d, f) show the specific heat capacity difference (*c*_p_ – *c*_p_^ref^) relative to the reference scan. The insets in panels (b-f) highlight the broad endothermic feature ascribed to the SCL transformation. To better highlight the first part of the annealing at *T*_a_ = 368.7 K, *c*_p_ – *c*_p_^ref^ curves in the inset of panel (f) were plotted differently, by placing shorter annealing times on top of longer ones.

Annealing at *T*_a_ = 338.7 K, that is, about 5 K below *T*_g_, induces physical aging^39,40^ of nonequilibrium glassy PLLA. As shown in Figure 2a, this is observed as a distinct endothermic peak emerging around 350–360 K, superimposed to the glass transition step. The *c*_p_ – *c*_p_^ref^ curve (Figure 2b) shows the characteristic shape of enthalpy recovery,^41^ with a negative exotherm peak slightly below 350 K followed by a more intense endotherm at higher temperatures. The negative contribution is commonly attributed to the upward shift of *T*_g_ resulting from the nonlinearity of physical aging,^42^ whereas the positive one is the signature of the rapid enthalpy increase to approach the SCL state and resulting from physical aging. The integration of *c*_p_ – *c*_p_^ref^ in the glass transition region provides the endothermic enthalpy recovery Δ*H*_rec_ from the glass to the liquid state. Apart from the standard transformation from glass to SCL, we detect the weak, broad endotherm in the SCL region (370–410 K), already observed by standard DSC and reported in Figure 1a Δ*H*_SCL_, appearing to saturate at the last annealing stages.

When the annealing temperature is increased above *T*_g_ (*T*_a_ = 353.7 K, Figure 2c), the endothermic overshoot characteristic of glass aging is suppressed. Nevertheless, supercooled liquid annealing effects persist. The *c*_p_ – *c*_p_^ref^ (Figure 2d) reveals a sharp negative peak slightly below 350 K, indicating a small upward shift in *T*_g_, accompanied by the arising of the low-intensity, broad endotherm spanning 370–420 K (Δ*H*_SCL_), already observed for annealing below *T*_g_. At longer annealing times, besides this broad endotherm, a high-temperature endothermic shoulder at 405 K appears, indicating the incipient melting of PLLA crystals formed during the annealing stage. Finally, annealing at temperatures above *T*_g_ (*T*_a_ = 368.7 K), that is, in the SCL state, results in a decreased intensity of the broad endotherm in the SCL state, followed by the typical pronounced thermal signature of PLLA melting (Figure 2e). From the *c*_p_ – *c*_p_^ref^ curves (Figure 2f), three distinct thermal events could be resolved: (i) a negative peak near 350 K, ascribed to an upward *T*_g_ shift; (ii) the broad, weak endotherm between 370 and 420 K, analogous to that seen at lower *T*_a_ but with faster evolution; and (iii) at longer annealing times, a sharp and intense endotherm at around 420 K, which is attributed to the melting of α′ crystals formed during annealing.^43,44^

To scrutinize the complex nonequilibrium behavior of the amorphous phases during annealing, the transformation kinetics of the three identified thermal events, which are physical aging, SCL transformation, and crystallization, were analyzed in detail. To this end, the enthalpy changes associated with these events were determined as a function of annealing time and temperature. Accordingly, the *c*_p_(*T*_a_,*t*_a_) – *c*_p_^ref^(*T*_a_,0) of each scan was integrated between 325 and 370 K to evaluate the enthalpy recovery (Δ*H*_rec_) employing eq 3, unequivocally associated with glass physical aging. Similarly, integration between 370 and 430 K was carried out to characterize the enthalpic change associated with the endothermic events occurring in the SCL phase (Δ*H*_SCL_), according to eq 4.

$$\Delta H_{\mathrm{rec}}(T_{a},t_{a})=\int_{325K}^{370K}\left[c_{p}(T_{a},t_{a})-c_{p}^{ref}(T_{a},0)\right]$$ 3

$$\Delta H_{\mathrm{SCL}}(T_{a},t_{a})=\int_{370K}^{430K}\left[c_{p}(T_{a},t_{a})-c_{p}^{ref}(T_{a},0)\right]$$ 4

In the latter case, Δ*H*_SCL_ at the longest annealing times is ascribed to both the SCL transformation and the melting of α’ crystals, the two processes being partially superimposed. The variations in Δ*H*_rec_ and Δ*H*_SCL_ as a function of *t*_a_ for all of the investigated *T*_a_ are shown in Figure 3a,b, respectively. Additionally, the equilibrated enthalpy values, reached at the end of glass physical aging and the SCL transformation, Δ*H*_eq,rec_ and Δ*H*_eq,SCL_, respectively, are displayed in Figure 3c.

**Figure 3:**
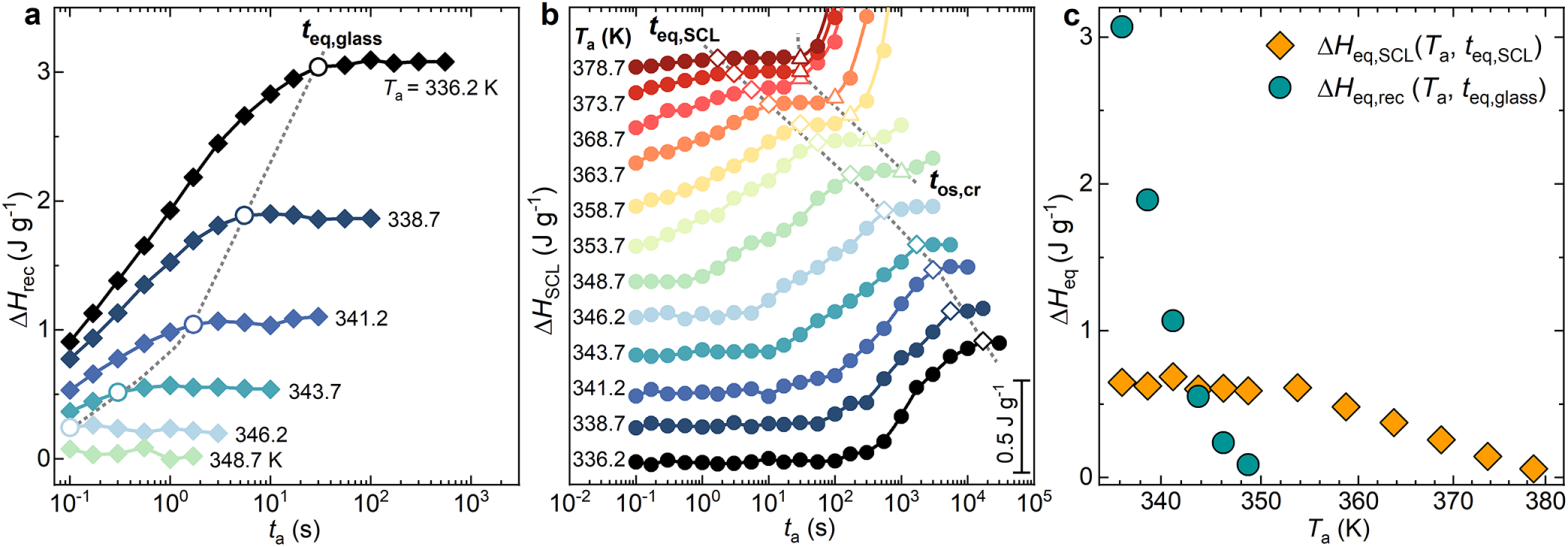
(a) Glass physical aging (Δ*H*_rec_) and (b) SCL transformation (Δ*H*_SCL_) enthalpic evolution as a function of annealing time at different annealing temperatures. Δ*H*_SCL_ in panel (b) is vertically shifted for clarity. Equilibration times of glass aging (*t*_eq,glass_) were visualized as open circles, equilibration times of SCL transformations (*t*_eq,SCL_) were visualized as open diamonds, and crystallization onset (*t*_os,cry_) was visualized as open triangles. Dotted lines are just a guide to the eyes to follow the temperature dependency for the three characteristic times. (c) Equilibrated enthalpy recoveries for glass aging Δ*H*_eq,rec_ and for the SCL transformation Δ*H*_eq,SCL_ as a function of *T*_a_.

As expected, in sub-*T*_g_ annealing experiments, Δ*H*_rec_ exhibits the typical behavior of glassy relaxation.^45^ A decrease in annealing temperature results in an increase in both the maximum recovered enthalpy Δ*H*_eq,rec_ (green circles in Figure 3c) and the time required to reach the steady-state value *t*_eq,glass_ (open circles in Figure 3a). This behavior is attributed to the nonequilibrium dynamics and thermodynamics of the glassy phase. Indeed, at annealing temperatures close to the experimentally observed *T*_g_ = 343 at 1000 K s^–1^, the recovered enthalpy becomes zero, the kinetics of physical aging being fast enough to maintain the supercooled liquid equilibrium state during cooling.

More complex is the time evolution of Δ*H*_SCL_. After an initial delay of the SCL transformation onset, which is more pronounced at lower *T*_a_, the Δ*H*_SCL_ values reached the stationary condition at *t*_eq,SCL_ (indicated as open diamonds in Figure 3b). Beyond this point, the further increase in Δ*H*_SCL_ observed at *T*_a_ > *T*_g_ can be ascribed to the incipient melting of the α’ crystalline phase. The annealing time at which the first traces of crystal formation are observed is denoted as *t*_os,cr_ and is shown as a series of open triangles in Figure 3b. As shown in Figure 3c, the enthalpy of the SCL transformation, Δ*H*_eq,SCL_, remains constant at 0.6 J g^–1^ up to approximately 353 K. Above this temperature, Δ*H*_eq,SCL_ decreases linearly, reaching a zero value at approximately 380 K.

Understanding the supercooled liquid (SCL) transformation requires a comprehensive examination of four key aspects: (i) the interplay among physical aging, SCL transformation, and crystallization; (ii) the thermodynamic stability of the supercooled liquid state; (iii) the influence of the SCL transformation on liquid dynamics; and (iv) the molecular mechanisms ruling the SCL transformation. These topics are discussed in detail in the following sections.

### Interplay between Physical Aging, SCL Transformation, and Crystallization

To further investigate the transformation occurring during PLLA isothermal annealing, its nucleation kinetics was studied by a two-stage Tammann protocol. This specific protocol has been extensively used for several polymers, including PLLA.^8,24,25^ As an example, the FSC heating curves of PLLA after a first nucleation stage at *T*_a_ = 368.7 K for different time *t*_a_ are displayed in Figure 4a. Here, the rise of the melting endotherm between 425 and 450 K was caused by the formation of crystal seeds at *T*_a_ that were successfully transferred at 398.7 K, leading to crystal growth. The use of a transfer rate of 100 K s^–1^ between the two isothermal stages allowed full preservation of the nuclei,^25^ enabling the direct relation between nuclei formation at *T*_a_ and crystal growth at *T*_c_. The melting enthalpies (Δ*H*_m_), determined for each explored annealing temperature (338.7 K ≤ *T*_a_ ≤ 378.7 K) and *t*_a_, are reported in Figure 4b. The annealing time at which Δ*H*_m_ departs from its base value has been employed to determine the onset of nuclei formation (*t*_os,nuc_, open stars in Figure 4b).

**Figure 4:**
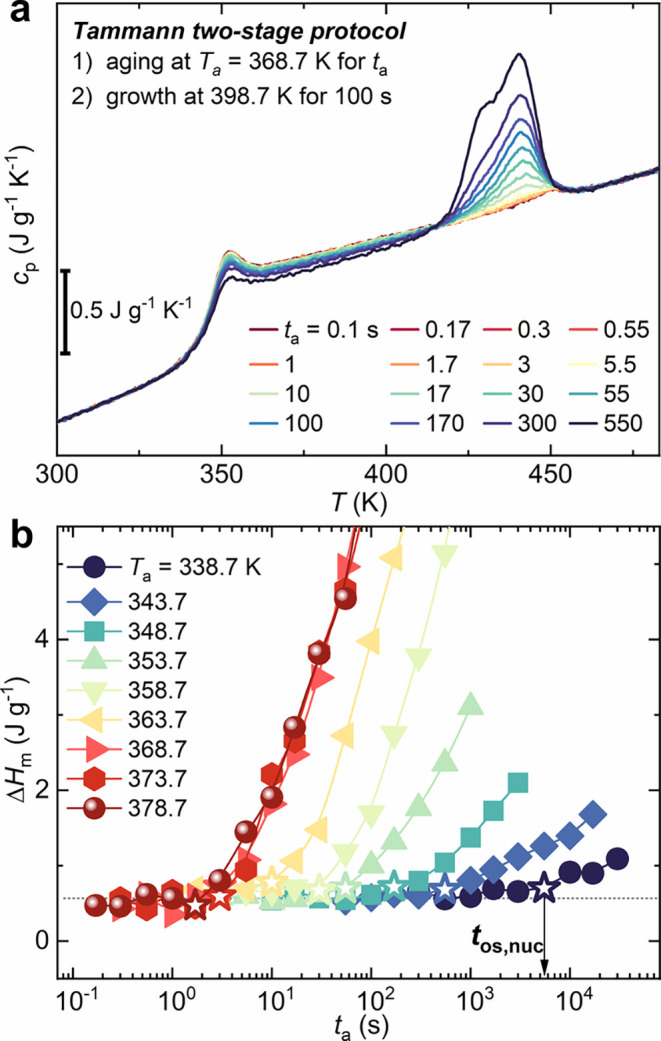
Two-stage Tammann protocol for PLLA. (a) FSC heating curves of PLLA samples subjected to a nucleation stage at *T*_a_ = 368.7 K for different times of *t*_a_, followed by a crystallization growth stage at 398.7 K for 100 s. (b) Enthalpy of fusion (Δ*H*_m_) as a function of annealing time for various nucleation temperatures. The intersection point with the baseline, shown with empty stars, represents the time of nuclei formation (*t*_os,nuc_).

Given these results, a complete scenario for transformation kinetics from amorphous glassy PLLA to the crystalline state can be built. The characteristic times of the four investigated thermal events, which are the glass physical aging offset *t*_eq,glass_, the SCL transformation offset *t*_eq,SCL_, the nucleation onset *t*_os,nuc_, and the crystal growth onset *t*_os,cr_, are summarized in the activation plot of Figure 5.

**Figure 5:**
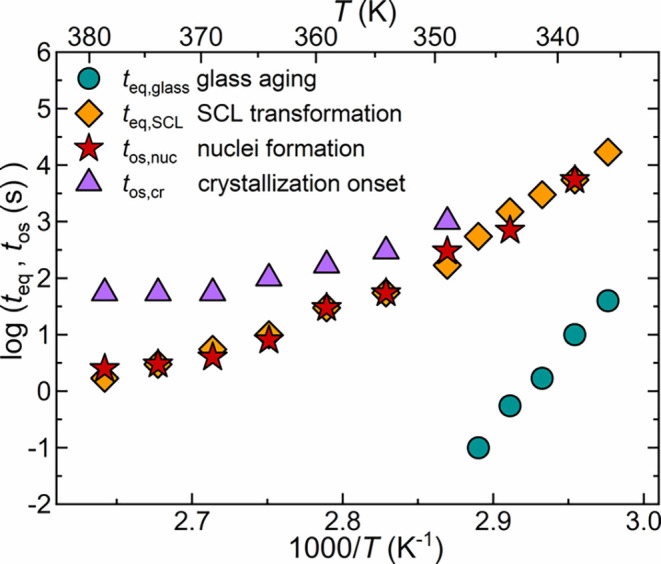
Activation plot for PLLA transformation kinetics. Equilibration time of glass aging (*t*_eq,glass_), equilibration time of SCL transformation (*t*_eq,SCL_), nuclei formation onset (*t*_os,nuc_), and crystallization onset (*t*_os,cr_) as a function of the inverse annealing temperature. The maximum uncertainty on logarithmic time scales is ±0.2, roughly corresponding to symbols size.

Calorimetric data reveal initial insights into the transformations occurring in the PLLA amorphous phase before crystallization. Specifically, a hierarchical relaxation pathway emerges, with the following sequence: *t*_eq,glass_ < *t*_eq,SCL_ ≈ *t*_os,nuc_ < *t*_os,cr_. The glass physical aging is the fastest kinetic transformation, enabling the glass to dissipate its excess enthalpy through structural rearrangements until reaching the extrapolated value of the SCL at the equilibration time designated as *t*_eq,glass_. Subsequently, the relaxation process in the SCL state (SCL transformation) occurs, characterized by equilibration times corresponding to *t*_eq,SCL_. This indicates that the relaxation involving a transformation within the SCL state proceeds on longer time scales. Interestingly, the onset time of nuclei formation nearly coincides with that at the end of the SCL transformation (*t*_eq,SCL_ ≈ *t*_os,nuc_). Ultimately, the first traces of crystalline growth are observed at *t*_os,cr_. This result hints at an intimate link between the SCL transformation and crystal nucleation.

### Thermodynamic Stability of PLLA Supercooled Liquid State

In this section, the enthalpy landscape of PLLA is analyzed and discussed in terms of the enthalpy deviation (*H*(*T*) – *H*_SCL_(*T*)) with respect to the supercooled liquid enthalpy, *H*_SCL_(*T*), extrapolated from a reference PLLA cooling scan at −1000 K s^–1^ according to Equations S1–4 in the SI. Detailed information about the mapping of the enthalpy landscape of amorphous PLLA is given in the “Enthalpy mapping of quenched and annealed PLLA” section of the SI. The primary focus is to check the enthalpy content and the stability of the supercooled liquid (SCL) resulting from the annealing isotherms.

Figure 6a illustrates the enthalpy deviations from the reference SCL state (*H*(*T*) – *H*_SCL_(*T*)), corresponding to the quenched SCL, after the annealing protocols were established at *T*_a_. In particular, the enthalpy deviation recorded during cooling at −1000 K s^–1^ is represented by the black line, establishing the initial enthalpy at the beginning of the annealing protocols. Green circles correspond to the enthalpy deviation of the equilibrated glass (*H*_eq,glass_ – *H*_SCL_) at the end of glass physical aging, calculated from Equation S6. Orange diamonds represent the equilibrated enthalpy deviation reached at the end of the SCL transformation (*H*_eq,SCL_ – *H*_SCL_), determined by Equation S7.

**Figure 6:**
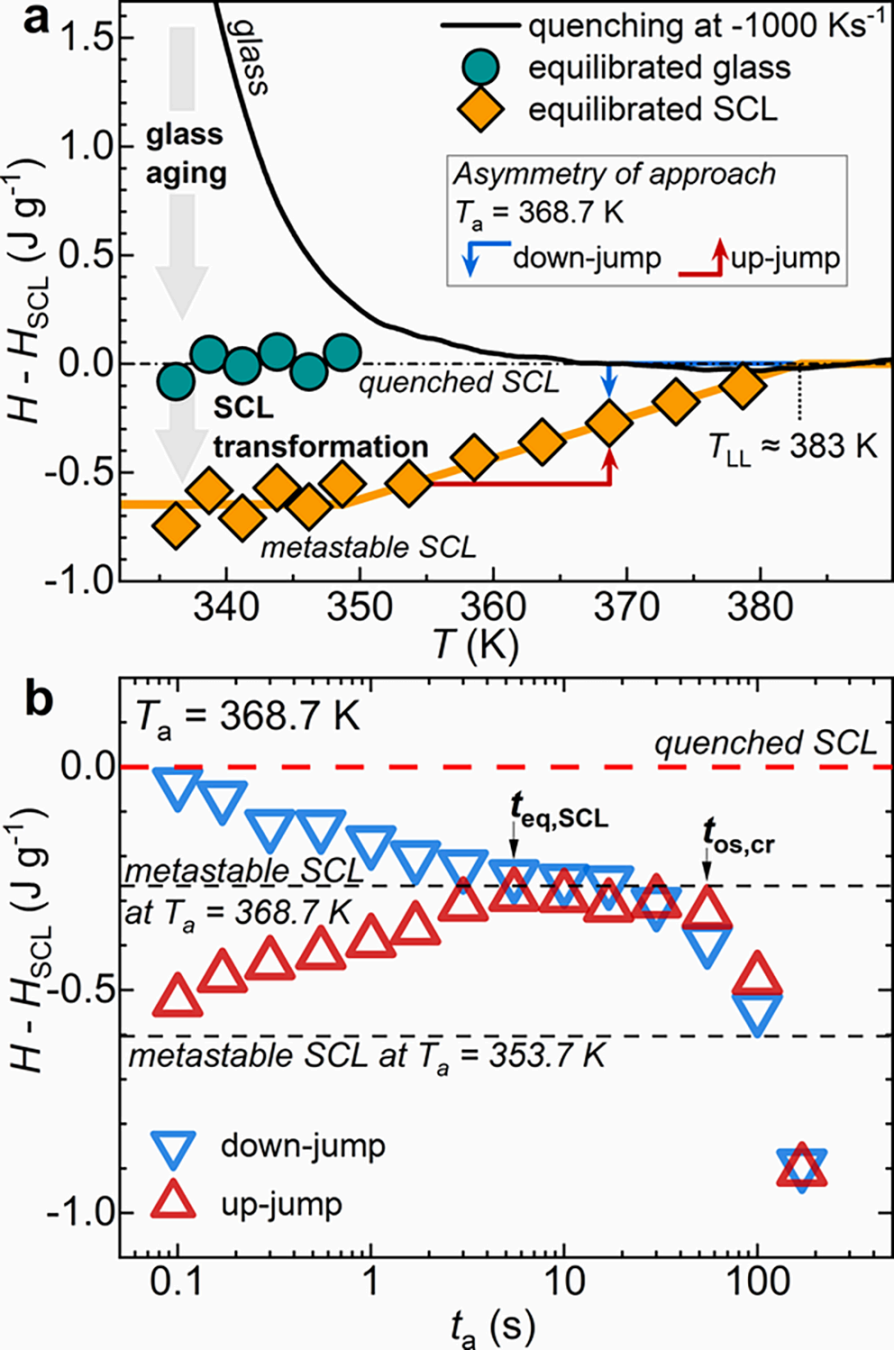
(a) Enthalpy deviation from the quenched SCL state, as a function of temperature. The black line represents the enthalpy deviation of PLLA quenched at −1000 K s^–1^, green circles represent the enthalpy plateau at the end of physical aging, and orange diamonds indicate the plateau value at the end of the SCL transformation. (b) Asymmetry of approach results, comparing the enthalpy deviation from the quenched SCL (*H* – *H*_SCL_) as a function of *t*_a_ for the up-jump (refer to Figure S4) and down-jump (refer to Figure 2e,f) for annealing at *T*_a_ = 368.7 K.

Physical aging of the nonequilibrium glassy state at *T*_a_ ≤ 348.7 K involves the dissipation of its enthalpy excess until it reaches the SCL enthalpy values from which the glass originates, i.e., the quenched SCL (*H*_eq,glass_ ≈ *H*_SCL_). After glass equilibration, the SCL transformation occurs, involving a further decrease in the enthalpy by an amount equal to −Δ*H*_SCL_(*T*_a_). As discussed in relation to Figure 3c, the SCL transformation led to an enthalpy loss of about 0.60 ± 0.05 J g^–1^ below *T*_a_ = 353.7 K. This gradually decreases with increasing temperature, reaching zero for *T*_a_ > 378.7 K. By linear extrapolation of *H*_eq,SCL_ – *H*_SCL_ for higher *T*_a_s, a characteristic temperature, defined as *T*_LL_ of 383 K, can be estimated as the temperature above which there is no longer enthalpy change caused by the SCL transformation.

To test whether the enthalpy state resulting from the SCL transformation represents a metastable state, the “asymmetry of approach” protocol was employed.^47–49^ This method verifies the stability of a thermodynamic state by checking if the system converges to the same final state when approached from different initial nonequilibrium conditions. If convergence to the same enthalpy plateau is observed, then the thermodynamic state under investigation can be considered stable or metastable. To apply this method effectively, an appropriate *T*_a_ must be selected where the system can be approached from both lower and higher enthalpy states. This condition is met within the annealing temperature range where the Δ*H*_eq,SCL_ value is intermediate (see Figure 3c). Therefore, the protocol was applied by approaching the same *T*_a_ = 368.7 K (Δ*H*_eq,SCL_ = 0.27 J g^–1^) from two different SCL states: a first standard annealing defined as “down-jump” started from the quenched SCL by simply cooling at 1000 K s^–1^ from the melt (*c*_p_ and *c*_p_ – *c*_p_^ref^ curves are displayed in Figure 2e,f; ΔH_SCL_ evolution is shown in Figure 3b); a second isothermal annealing defined as “up-jump” started from low enthalpy by a prior annealing at 353.7 K for 100 s before jumping to *T*_a_. A sketch of the expected *H*(*T*) trajectories for down- and up-jump experiments is given by the blue and red arrows in Figure 6a, respectively. The evolution of *H* – *H*_SCL_ with annealing time for the two experiments is shown in Figure 6b, calculated from the *c*_p_ – *c*_p_^ref^ curves of Figure S4. The down- and up-jump enthalpy evolutions reveal that the approach to the common plateau at *H*_eq,SCL_ – *H*_SCL_ = −0.27 J g^–1^ is symmetrical and simultaneous, occurring at *t*_eq,SCL_ = 5.5 s. The symmetry of the approach to equilibrium is uncommon in glasses, where a marked asymmetry is found instead, caused by the nonlinearity of glass relaxation.^48^ Noteworthy, both approaches gave the same crystallization onset, excluding the formation of nuclei during the preannealing treatments during the up-jump protocol.

Considering these results, the SCL transformation appears as an additional kinetic process, occurring on time scales several orders of magnitude slower than those associated with the main α-relaxation, assisting the standard supercooled liquid to reach a lower free energy metastable state. Specifically, for PLLA cooled at 1000 K s^–1^, the main structural α-relaxation fails to drive the SCL to this lower free energy metastable equilibrium for temperatures below *T*_LL_ = 383 K. As a consequence, the quenched SCL state of PLLA below 383 K can be classified as an unstable state, prone to spontaneous evolution toward a metastable SCL state through the SCL transformation process.

### Influence of SCL Transformation on Segmental Dynamics

As previously discussed, both SCL transformations brought about a subtle *T*_g_ upward displacement. Figure S5 of the SI shows a magnification of the *c*_p_ heating scans in the *T*_g_ range for all annealing temperatures and times explored in the current study.

Figure 7a displays the evolution of the *T*_g_ displacement (*T*_g_ – *T*_g_^ref^), relative to *T*_g_^ref^, that is, the *T*_g_ of the scan obtained immediately after cooling from the melt at 1000 K s^–1^, as a function of *T*_a_ and *t*_a_. A distinct bimodal increase is observed for annealing in the glass (*T*_a_ < 348.7 K), whereas a monotonic trend is observed for SCL annealing (*T*_a_ ≥ 348.7 K).

**Figure 7:**
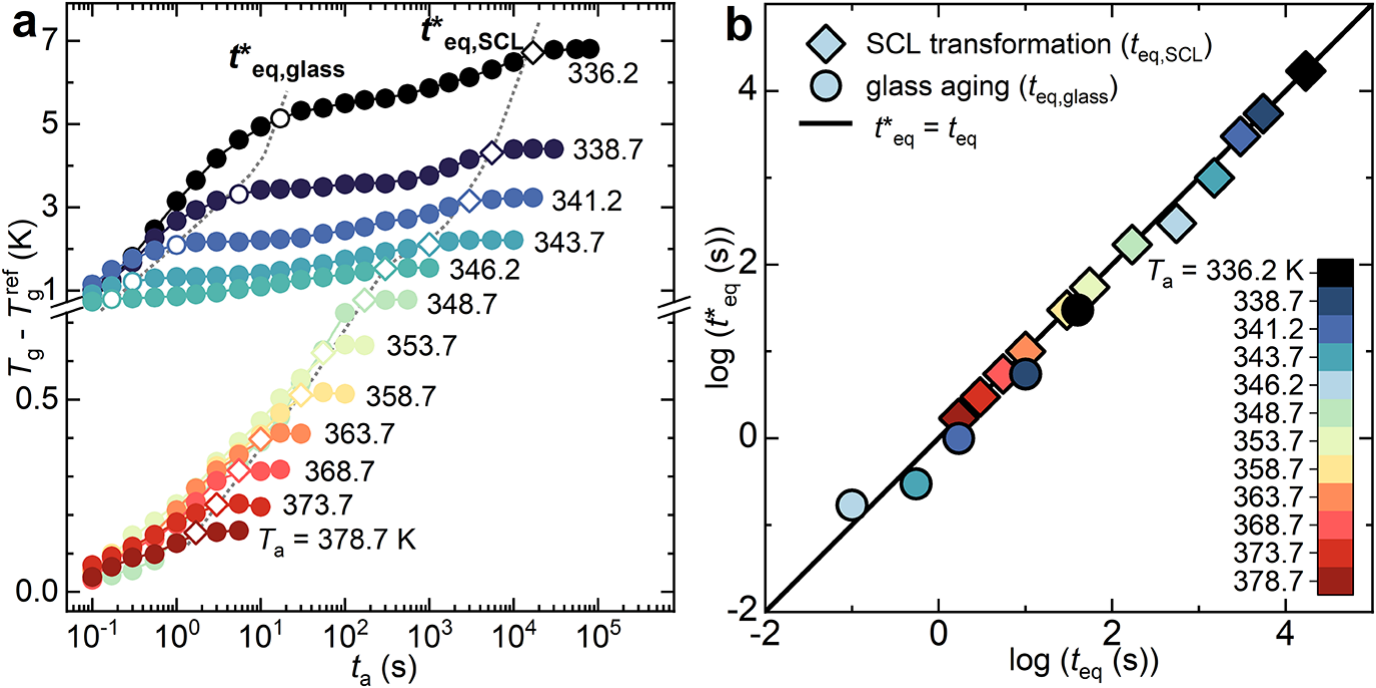
(a) Evolution of the *T*_g_ displacement between annealed and reference scans as a function of *t*_a_ at different annealing temperatures. The *T*_g_ shift was determined at the inflection point of the specific heat step. Equilibration time of glass aging (t*_eq_,_glass_) and equilibration time of the SCL transformation (t*_eq_,SCL) are represented as open circles and open diamonds, respectively. The dotted lines are guides for the eyes. (b) Correlation plot between the equilibration times determined from enthalpy stabilization (*t*_eq_, reported in Figure 3a,b) and from the *T*_g_ displacement plateau (t*_eq_). The correlation was checked for both glass annealing and SCL transformation at all of the explored *T*_a_.

The faster kinetic evolution of the glass, intrinsically linked to its nonequilibrium nature as it approaches the quenched SCL state, is characterized by a positive *T*_g_ displacement of 1–5 K depending on *T*_a_ and *t*_a_. Similar to the enthalpy recovery kinetics (Figure 3a), this process terminates at a characteristic time, denoted as t*_eq,glass_, as indicated by the slope change represented as open circles in Figure 7a. This *T*_g_ increase is ascribed to the “nonlinearity” of standard physical aging of glasses. The latter implies that the thermodynamic state of a glass, in terms of enthalpy and volume, influences its α-relaxation time. As the glass undergoes enthalpy decrease and density increase during physical aging, its α-relaxation time decreases, leading to a positive shift in *T*_g_.^48,49^ Nevertheless, the slower and less intense increase in *T*_g_, stabilizing at annealing time *t* ^*^_eq,SCL_ (open diamonds in Figure 7a), cannot be addressed considering the nonlinearity of standard glass physical aging. Based on the experimental evidence presented in the previous section, it can be inferred that the *T*_g_ displacement could be caused due to the enthalpy loss Δ*H*_SCL_ associated with the SCL transformation, resulting in a slight slow-down of PLLA segmental mobility. Reinforcing this hypothesis, an almost 1:1 correlation between the equilibration times retrieved from enthalpy evolution (*t*_eq_,_glass_, *t*_eq_,_SCL_ from Figure 3a,b) and from the *T*_g_ displacement (t*_eq,glass_, t*_eq,SCL_ in Figure 7a) is found. The correlation plot between these characteristic times is presented in Figure 7b.

Beyond the transformation kinetics described above, subtle variations in PLLA segmental dynamics caused by the SCL transformation were discerned by a step-response protocol,^30,50,51^ conveying information on the spontaneous fluctuations of the system in the linear response regime.^45^

Figure 8a reports the real part of the complex specific heat, *c*_p_^′^(ω, *T*), normalized between 0 (glass) and 1 (liquid), for five exemplificative frequencies of a quenched and an annealed PLLA at *T*_a_ = 353.7 K for 100s. The annealing brought a decrease in an enthalpy of 0.6 J g^–1^ with respect to the quenched PLLA (see Figure 3c).

**Figure 8:**
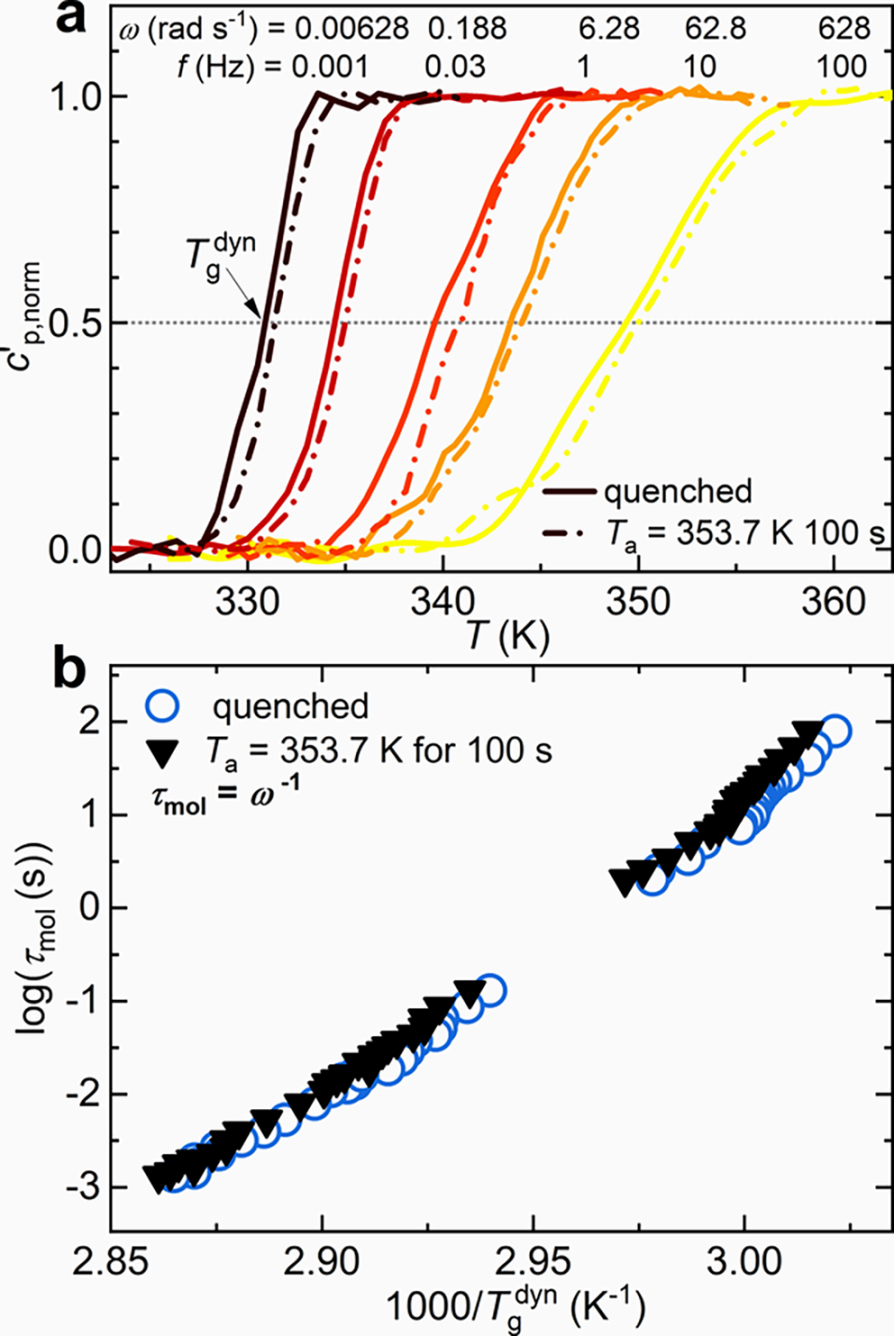
(a) Frequency-dependent real part of the normalized complex specific heat, *c*_p_^′^(ω, *T*), for quenched PLLA and PLLA annealed at 353.7 K for 100 s. The measurements are presented at five different frequencies, namely, 0.001, 0.03, 1, 10, and 100 Hz. (b) Temperature dependence of the segmental relaxation time for both quenched and annealed PLLA samples, illustrating the differences in dynamics resulting from the SCL transformation.

Figure 8a shows that the temperature at the half step in *c*_p_^′^(ω, *T*), i.e., the dynamic glass transition measured in the linear response, *T*_g_^dyn^, lays systematically at higher temperatures for the annealed PLLA. By plotting the characteristic time of segmental relaxation τ_mol_ = ω^–1^ = (2π*f*)^−1^ against the inverse of *T*_g_^dyn^, the temperature dependence of PLLA segmental dynamics is retrieved (see Figure 8b). In the whole τ_mol_ range, the *T*_g_^dyn^ difference between quenched and annealed PLLA lies between 0.5 and 1.5 K, which is compatible with the *T*_g_ shift of 0.65 K obtained after completion of the SCL transformation at 353.7 K (see Figure 7a). These results confirm that the SCL transformation slightly hampers segmental mobility, thus anticipating that the enthalpic losses could be related to local chain rearrangements in the liquid, occurring before PLLA nucleation. The microstructural aspects of the SCL transformation are discussed in the next section.

### Conformational Facets of the SCL Transformation

To investigate the molecular mechanisms underneath the SCL transformation, which has thus far been evidenced solely through calorimetric techniques, an analysis of the conformational evolution of PLLA polymer chains during annealing in the *T*_g_ range was performed by time-resolved Fourier-transform infrared spectroscopy (TR-FTIR). Indeed, the PLLA vibrational spectrum, being sensitive to different physical and chemical features of the polymeric chain, has been extensively studied to investigate its thermal transitions.^27,52,53^ The repeating unit of PLLA is characterized by two degrees of rotational freedom around the two skeletal C–C and C–O bonds, leading to four stable rotational isomers, or conformers, defined as *gg*, *gt*, *tg*, and *tt*, depending on the dihedral angle of the two bonds.^54^ The *gt* conformer has the lowest energy and, if repeated along the chain, forms the 10_3_ helices of the crystal lattice. On the other hand, the high-energy *gg* conformation is predominant in amorphous PLLA. In general, *gg* and *gt* forms contribute for more than 90% of the overall conformational population, and a conformational equilibrium between them is established in the liquid phase.^55^ Moreover, the transformation from *gg* to *gt* and vice versa constitutes the molecular mechanism underneath the change in macroscopic properties during phase transitions. Therefore, by following the intensity changes of selected absorption bands during isothermal annealing, the microstructure of PLLA chains could be determined. Within the scope of the present work, the intensities of three characteristic absorptions at 1267, 957, and 922 cm^–1^ were studied. In particular, the band at 1267 cm^–1^ is assigned to the ν_as_(COC) + δ­(CH) vibrational mode of the *gg* conformer, while those at 957 and 922 cm^–1^, arising from the same r­(CH_3_) + ν­(C–COO) coupled vibration, are associated with the formation of the 10_3_ helix (crystalline phase) and with disordered chains (amorphous phase), respectively. The ratio between the 922 cm^–1^ intensity (*A*_922_), divided by the sum of the 922 and 957 cm^–1^ intensities (*A*_922_ + *A*_957_), has been exploited as a crystallinity index, a way to determine its fraction *X*_c_.^56^ Figure 9 presents time-resolved infrared spectra of amorphous PLLA obtained at *T*_a_ = 331.5 and 346.8 K for selected annealing times.

**Figure 9:**
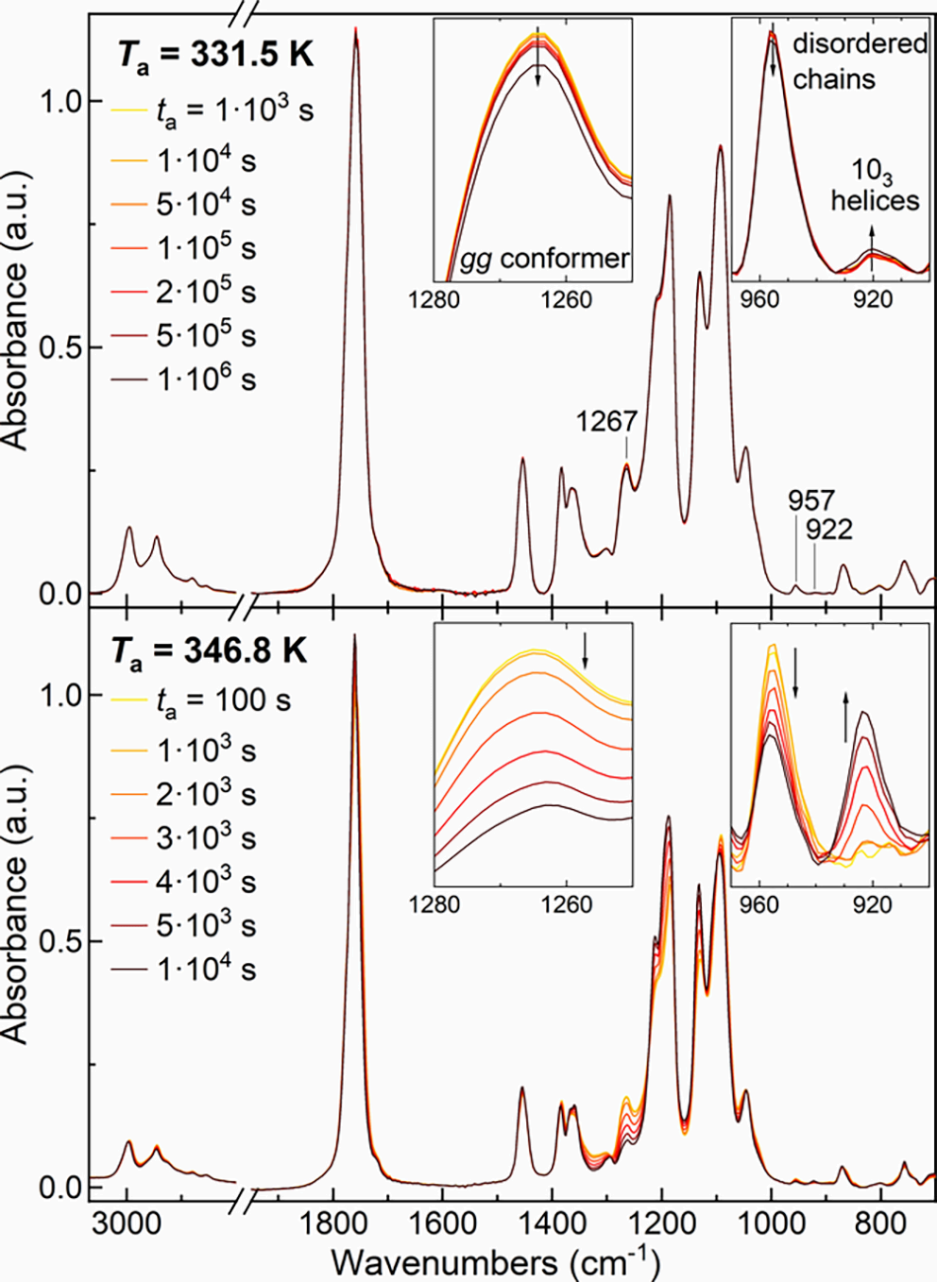
FTIR spectra of amorphous PLLA annealed at 331.5 K (upper panel) and at 346.8 K (lower panel) for different *t*_a_. The insets highlight the intensity changes in the bands at 1267, 957, and 922 cm^–1^.

The observed spectral changes indicate the evolution of the amorphous phase during annealing at two indicative temperatures. At the lower *T*_a_ = 331.5 K, the 1267 cm^–1^ absorption undergoes a subtle, gradual decrease up to 500000 s, due to the SCL transformation, followed by an intensity drop at 1 × 10^6^ s. At the same time, the intensities of the peaks at 957 and 922 cm^–1^ remain stable up to 500000 s, after which the rise of the 922 cm^–1^ band highlights the incipient crystallization. Conversely, the higher *T*_a_ of 346.8 K favors cold-crystallization kinetics. In this case, a substantial decrease of the 1267 and 957 cm^–1^ bands and a simultaneous rise of the 922 cm^–1^ band are observed.

To highlight the spectral evolution of PLLA during annealing at all of the explored temperatures, the crystallinity index, *X*_c_, and the normalized intensity at 1267 cm^–1^ (*A* ^1267^/*A*_0_^1267^) are shown as a function of *T*_a_ and *t*_a_ in Figure 10.

**Figure 10:**
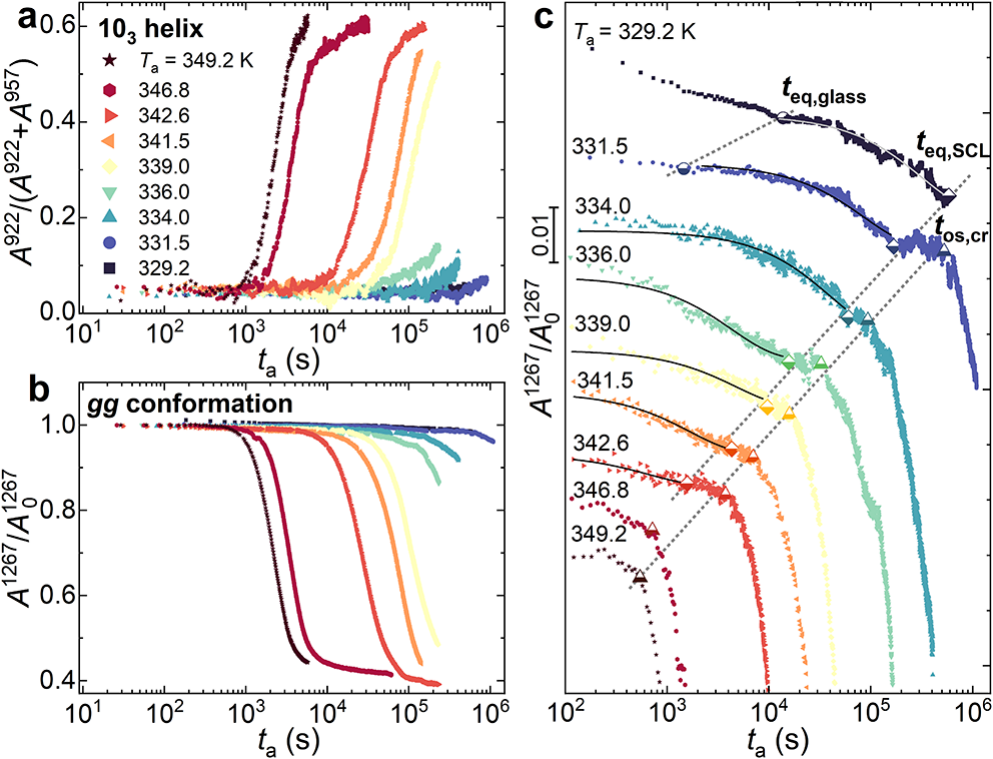
(a) Crystallinity index, calculated as *A* ^922^/(*A* ^922^ + *A* ^957^) of PLLA as a function of *t*_a_ at various *T*_a_. (b) Normalized intensity of the absorption band at 1267 cm^–1^ as a function of *t*_a_ for different annealing *T*_a_. (c) Magnification of *A* ^1267^/*A*_0_^1267^ before the onset of crystallization as a function of *t*_a_ for different annealing *T*_a_. The curves are vertically shifted for clarity.

At first glance, the crystallization onset *t*_os,cr_ is observed in correspondence to the departure of *X*_c_ from the base value of 0.05, caused by the presence of residual helical structures in the amorphous phase.^46^ Conversely, the intensity of the normalized band at 1267 cm^–1^ shows an opposite trend due to the *gg* population transformation in low-energy *gt* conformers, building the helix structures and, ultimately, the crystal lattice.

Through a more accurate inspection of the *A* ^1267^/*A*_0_^1267^ evolution before crystallization occurs, that is, when the sample is in the glassy or SCL state, the change of *gg* concentration during the first annealing stages can be accessed. Figure 10c reports a magnification of the first annealing parts of the *gg* evolution reported in Figure 10b, vertically shifted for clarity. Interestingly, two distinct concentration decays emerge prior to the onset of crystal formation (*t*_eq,os_, half-filled triangles in Figure 10c), which are physical aging, for *T*_a_ = 329.2 and 331.5 K, and the SCL transformation, visible for annealing temperatures up to *T*_a_ = 342.6 K. Starting from low annealing temperatures, a fast decay is observed up to the stabilization at *t*_eq,glass_ (half-filled circles in Figure 10c). This is related to the physical aging process, implying a structural reorganization of a small moiety of the high-energy *gg* conformers into energy-favorable *gt* conformers.^57^ Following this, a second decay of *gg* concentration is observed between the end of physical aging and the onset of crystallization, indicating a conformational rearrangement of the SCL phase. The latter proceeds following approximately a simple exponential decay law (black lines in Figure 10c) to reach a steady *gg* population at *t*_eq,SCL_ (half-filled diamonds), which decreases as the annealing temperature increases. The magnitude of the conformational transition in the SCL can be estimated from the amplitude of the exponential decay, resulting in an average loss equal to 1.7 ± 0.3% of the initial *gg* population. Indeed, the *gg* conformer is converted into *gt* low-energy conformers without provoking detectable formation of helical structures.

### Origin of SCL Transformation: The Case of Poly­(D,l-lactide)

To unveil whether the SCL transformation originates from the ability of PLLA to crystallize or it is an enthalpic relaxation involving just the presence of multiple supercooled liquid states, annealing measurements were performed on a PLLA analogue, poly­(D,l-lactide) (PDLLA). PDLLA differs from PLLA as it possesses a disordered configurational sequence of D- and L- stereoisomers along the chain (in contrast to PLLA’s isotactic L-configuration). The configurational disorder hinders the formation of regular helical structures^46^ and, ultimately, prevents chain packing into the crystal lattice. Figure 11 reports the heat capacities *C*_p_ and heat capacity differences *C*_p_ – *C*_p_^ref^ during heating at 1000 K s^–1^ for PDLLA annealed in the glass (*T*_a_ = 323.2 K) and in SCL (*T*_a_ = 353.2 K) phases for various annealing times (0.1 s up to 40,000 s). Similarly to PLLA, annealing in the glass led to physical aging, evidenced by a sharp overshoot peak superimposed to the *T*_g_ step. Conversely, the calorimetric signatures of the SCL transformation and those of melting are absent for both annealing temperatures.

**Figure 11:**
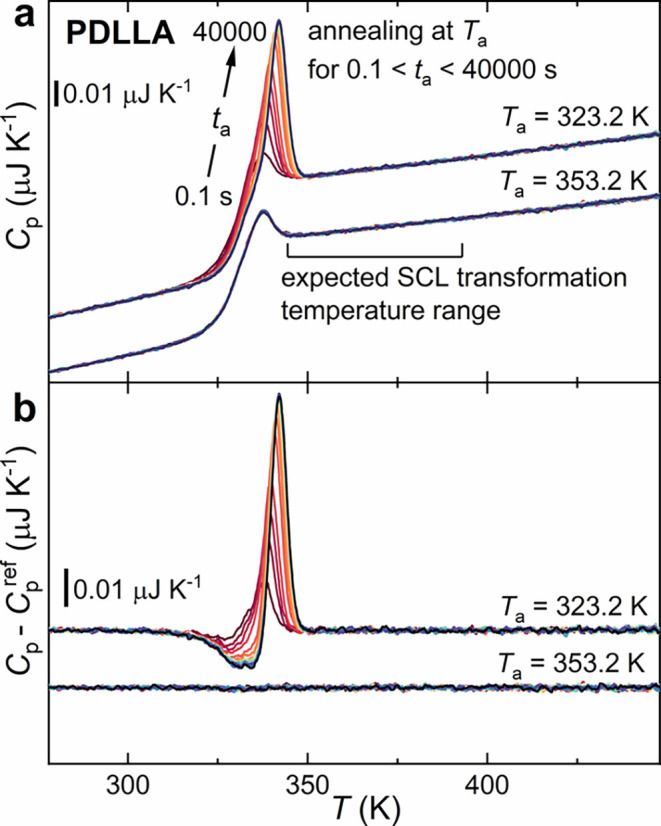
(a) *C*_p_ and (b) *C*_p_ – *C*_p_^ref^ heating curves for isothermal annealing of PDLLA at *T*_a_ = 323.2 and 353.2 K, for annealing times spanning 0.1–40,000 s.

In analogy to FSC measurements, the lack of the SCL transformation for PDLLA is further corroborated by time-resolved FTIR measurements. Figure 12 reports the time-dependent FTIR spectra of PDLLA recorded during annealing at *T*_a_ = 348.2 K, together with the time evolution of the normalized intensity of the 1267 cm^–1^ *gg* conformational band and of the crystallinity index *X*_c_ = *A* ^922^/(*A* ^922^ + *A* ^957^).

**Figure 12:**
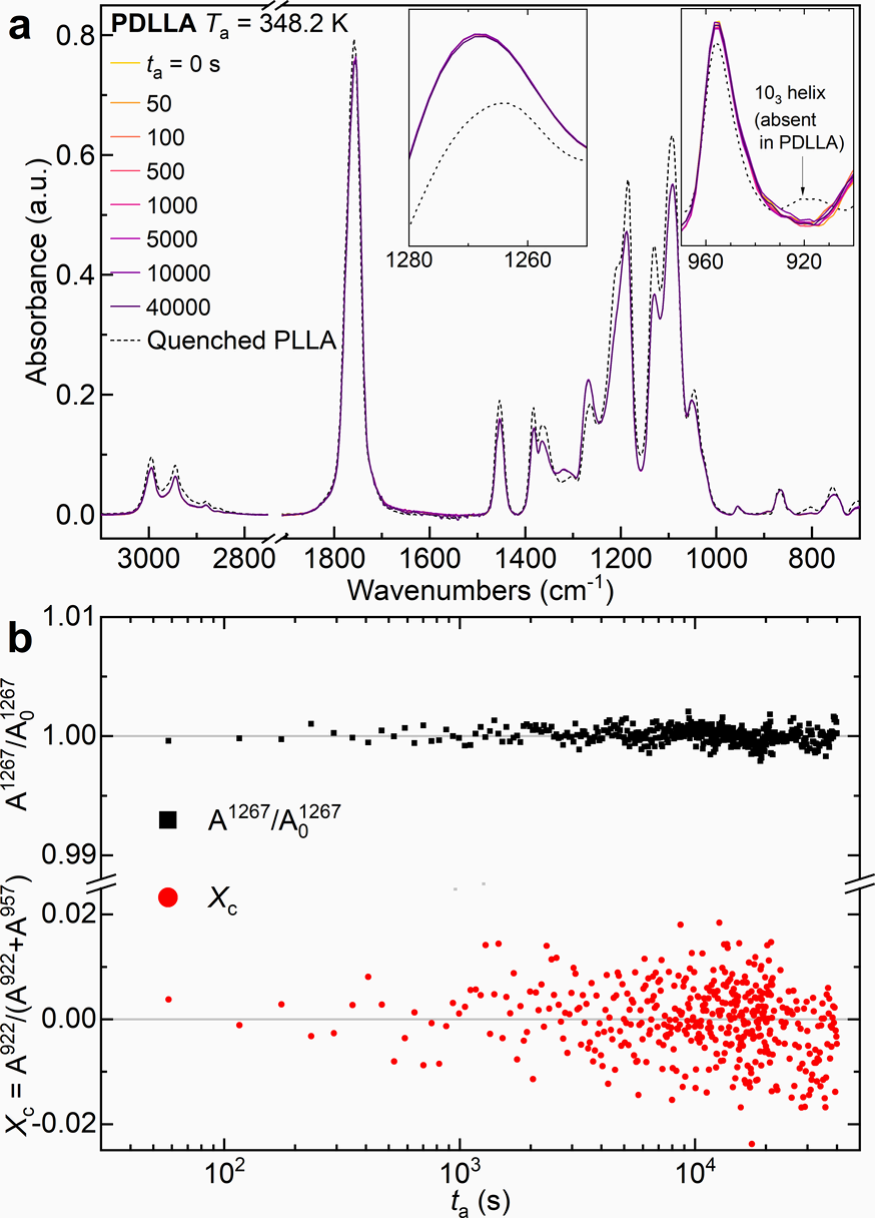
(a) FTIR transmission spectra of PDLLA annealed at 348.2 K for different times ranging between 0 and 40000 s. Insets magnify the 1267, 957, and 922 cm-1 absorption bands. For comparison, a spectrum of amorphous PLLA is reported in a dashed black line. (b) Intensity variations of the normalized 1267 cm^–1^ band and of the crystallinity index *X*_c_ as a function of annealing time.

At first glance, the IR spectrum of PDLLA (Figure 12a) is almost identical to that of PLLA, but the complete absence of the 922 cm^–1^ band is observed. As discussed before, the absence of this band must be sought in the inability of PDLLA to form helical structures. Along with the null variation of *X*_c_ during annealing, the 1267 cm^–1^ intensity variation also does not show any sign of SCL transformation through *gg-*to-*gt* conformational rearrangement (Figure 12b). These results confirm that, even at the microstructural level, the SCL transformation could exist only if the polymer could relax toward a more stable state than the liquid one, that is, the crystal. Given these observations, the SCL transformation seems to act as a preordering step toward crystal nucleation.

### Thermal and Conformational Mapping of PLLA Transformation Kinetics

In this final section, we combine spectroscopic and calorimetric information to depict an extensive overview of the transformation kinetics occurring in the amorphous phases of PLLA, from both microstructural and thermal viewpoints. In Figure 13, the equilibration time scales of glass relaxation and of the SCL transformation, *t*_eq,glass_ and *t*_eq,SCL_, the nucleation and crystallization onset, *t*_os,nuc_ and *t*_os,cr_, from FSC, DSC, and FTIR techniques, are compared with the segmental dynamics of quenched PLLA assessed calorimetrically by means of step-response analysis. To ensure a direct comparison between the characteristic time scales retrieved for nonequilibrium measurements with segmental relaxation time scales obtained by measurements in the linear regime, the latter were displaced by 1.5 orders of magnitude to align τ_mol,α_ with *t*_eq,glass_, in accordance with the Frenkel–Kobeko–Reiner approximation:^58^

$$\log\;t_{\mathrm{eq},\mathrm{glass}}=\log\;\tau_{\mathrm{mol}}+\log\;C$$ 5

**Figure 13:**
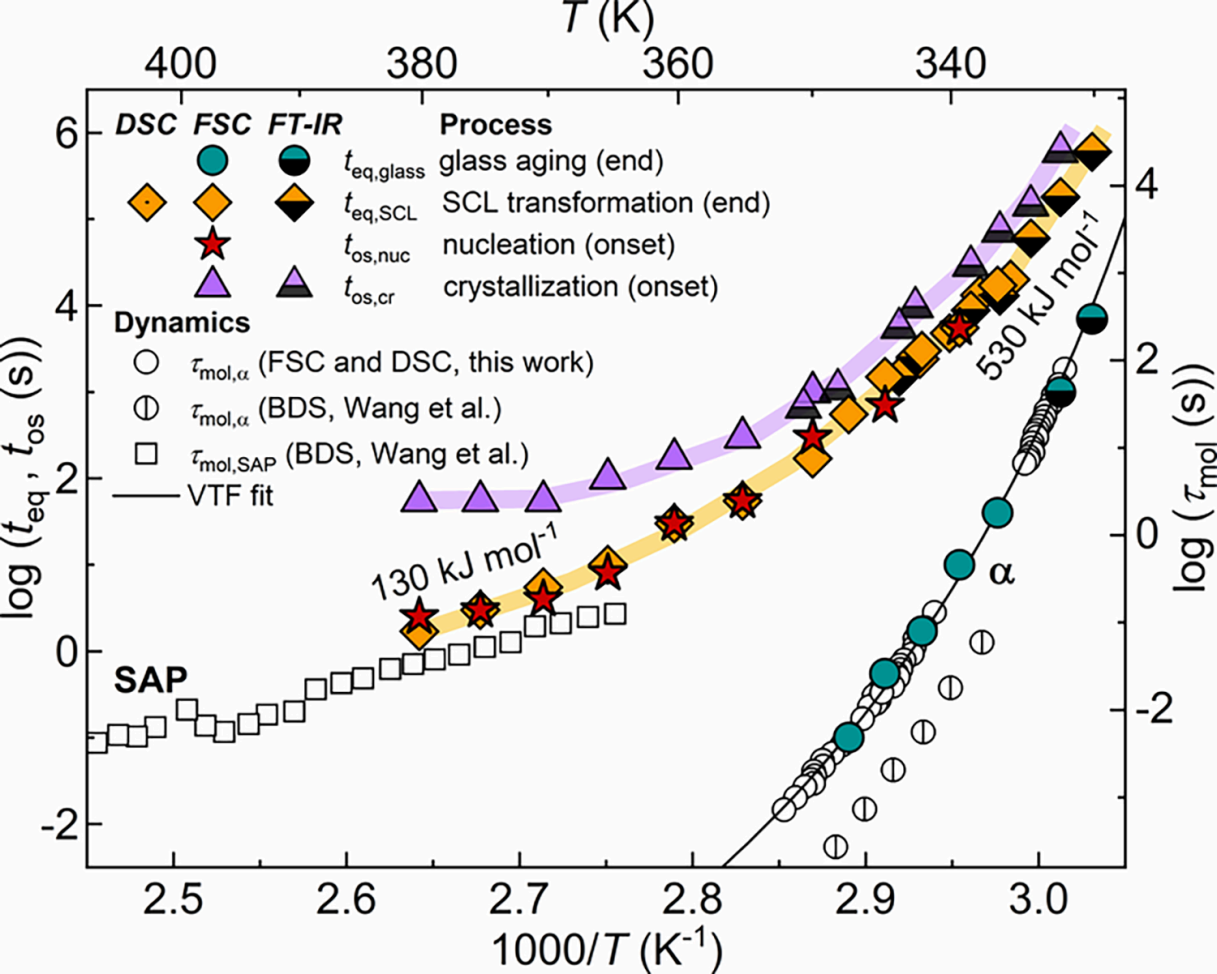
Activation plot of PLLA, obtained by FSC (full marks), DSC (dot-in-marks), and TR-FTIR (half-filled marks). Left axis: equilibration time of glass aging (*t*_eq,glass_), equilibration time of SCL transformation (*t*_eq,SCL_), nuclei formation onset (*t*_os,nuc_), and crystallization onset (*t*_os,cr_) as a function of the inverse temperature. The maximum uncertainty on logarithmic time scales is ±0.2, roughly corresponding to symbol size. Right axis: segmental relaxation time (τ_mol_) by the step-response protocol, molecular relaxation time of the main α-relaxation and that of the SAP process, taken from Wang et al.^33^

where *C* is the FKR coefficient, and log *C* = 1.5 in our case.

Figure 13 shows that the time scales of analyzed relaxations calculated from FTIR are in good agreement with the calorimetric ones, thereby bridging a link between microstructural information and enthalpy relaxation kinetics of amorphous PLLA (Figure 5). Glass physical aging is the fastest relaxation, whose equilibration kinetics exhibit marked super-Arrhenius behavior. Its temperature dependence is identical to that of the main α-relaxation, indicating that this relaxation is the main molecular mechanism, at least in the considered temperature regimes, controlling the spontaneous transformation from nonequilibrium glass to the SCL state from which the glass originated. Deviations from the VFT law may be observed for aging deep in the glassy state, as reported for several polymeric^59–62^ and nonpolymeric glasses,^50,63,64^ where other non-α-mechanisms compete with the α-relaxation to assist glass equilibration.

After reaching the unstable SCL state originated by quenching, an additional kinetic phenomenon takes place, that is, the SCL transformation, detectable below *T*_LL_ = 383 K (see Figure 6a). This kinetic event originates from the fact that the supercooled liquid obtained by fast quenching (1000 K s^–1^) is unable to maintain the metastable equilibrium properties below 383 K, leading to an unstable liquid with generally small enthalpy excesses, up to a maximum of 0.6 J g^–1^ (Figures 3c and 4a). The effects of the SCL transformation may reflect on mechanical properties, as already observed for PLLA, showing that rapid quenching (absence of SCL relaxation) results in brittle glasses.^32^ Conversely, annealing in the SCL state (329 ≤ *T*_a_ ≤ 353 K for 10 min) resulted in a huge increase of glass ductility and a subtle increase of *T*_g_.^32^ The latter agrees well with the decrease in segmental dynamics recorded after the completion of the SCL transformation at 353.7 K for 100 s, as shown in Figure 8. Concerning SCL relaxation kinetics, the almost perfect overlapping between the calorimetric time scales (full and dot-in-mark diamonds of Figure 13), and those of the conformational reorganization prior to crystallization by vibrational spectroscopy (half-filled diamonds in Figure 13, taken from Figure 10c) constitute an unprecedented result on the SCL transformation seen from different perspectives. Thus, it can be inferred that the conformational transition of a small fraction of the *gg* conformer into the low-energy *gt* form is the molecular mechanism underneath the small enthalpy loss during the SCL transformation. Since *gt* is the suitable conformer to build 10_3_ helices, which are the building blocks of α’ and α crystals,^65^ its population increase during the SCL transformation favors the crystallization of PLLA. Indeed, it has been shown that the first traces of nuclei were observed where the SCL transformation ends (*t*_eq,SCL_ ≈ *t*_os,nuc_). The apparent coupling between the SCL relaxation and nucleation kinetics seems crucial to understanding the driving force behind the SCL transformation. It is important to recall that at temperatures where SCL relaxation is observed (below 383 K), the thermodynamically stable equilibrium state for PLLA is the crystalline phase, up to its melting point. This suggests that the relaxation of the rapidly quenched SCL state could be a reorganization driven by the existence of the stable crystal state.

At the microstructural level, polymer chains tend to reorganize into ordered structures along the chain (medium-range order) that subsequently arrange spatially to form long-range crystalline order. Specifically, PLLA crystallization typically occurs when chain segments locally adopt energetically favorable *gt* sequences along the chain to form regular structures, that is, the 10_3_ helix.^66^ These ordered stems then aggregate by lateral stacking, incorporating chain folds at the growth front, to form stable crystalline lamellae.^67^ Indeed, the conformational rearrangement within the SCL state of PLLA appears to be a plausible precursor step, facilitating the sequential ordering of *gt* conformers into helical structures at the lamellar growth front. This could suggest that the driving force for the SCL transformation is intrinsically linked to PLLA’s potential to crystallize.

To test this hypothesis, isothermal annealing experiments at two *T*_a_ (below and above *T*_g_) were performed using FSC and TR-FTIR on poly­(D,l-lactide) (PDLLA), the PLLA analogue that was unable to crystallize. Figures 11 and 12 clearly show that PDLLA exhibits just standard glass physical aging, whereas no SCL transformation or crystallization is detected. This result supports the hypothesis that the SCL transformation observed in PLLA is intrinsically linked to, and likely driven by, the polymer's capability to organize into more ordered structures, a pathway unavailable to the noncrystallizable PDLLA.

The temperature dependence of the SCL transformation was determined as the slope of time scales as a function of inverse temperature, as shown in Figure 13. The activation energy weakens with increasing temperature, starting from a value of 530 ± 20 kJ mol^–1^ at *T*_a_ close to *T*_g_ up to 130 ± 20 kJ mol^–1^ at the higher temperatures where the SCL transformation can be observed.

To gain insight on the underlying molecular mechanisms mediating the SCL transformation, the associated activation energy is compared to PLLA liquid dynamics observed by Broadband Dielectric Spectroscopy (BDS).^33^ Beyond the cooperative α-relaxation, Song et al. recently discovered an additional noncooperative relaxation, addressed as the Slow Arrhenius Process (SAP),^34^ characterized by Arrhenius temperature dependency and slower dynamics above *T*_g_ with respect to the α-relaxation. In the last years, accumulating evidence points toward the SAP being a universal process characterizing amorphous materials, including polymers^34^ and molecular glasses.^68^ It has been demonstrated that the SAP is responsible for several equilibration mechanisms, occurring both in the liquid and in the glassy state, including polymer melting,^33^ polymeric chain adsorption and dewetting,^69–71^ and glass equilibration deep in the glassy state.^59,60,63,64,72^ For a selection of polymers, including PLLA, the SAP activation energy (*E*_SAP_) is almost equal to the activation energy of the viscous flow in the melt state, *E*_flow_, which is the energy barrier required for the chain segments of a polymer to overcome friction against neighboring molecules and transit to nearby voids. For PLLA, *E*_SAP_ and *E*_flow_ exhibit values of 95 ± 10 and 91 ± 5 kJ mol^–1^,^33,73^ respectively, which are comparable to the activation energy of the SCL relaxation at high temperatures, *E*_SCL_ = 130 ± 20 kJ mol^–1^. Additionally, Figure 13 puts in relation the time scales assessed for the SCL transformation and for the SAP mechanism, clearly testifying a close relation between the SCL equilibration time scales and those of the SAP process at high temperatures.^33^ Given that the SCL transformation takes place in the supercooled liquid, it is reasonable to infer that the SCL transformation’s kinetics are influenced by both the SAP and the α-relaxation, with its rate being limited by the slowest dynamics. Indeed, at high temperatures, where the SAP time scale is significantly longer (by >5 orders of magnitude) than the α-relaxation time scale, the activation energy of the SCL transformation (∼130 kJ mol^–1^) approaches that associated with the SAP or viscous flow (∼95 kJ mol^–1^). Conversely, when the temperature is decreased, the increasing influence of the α-relaxation results in a much steeper temperature dependence compared to the SAP. This causes the α-relaxation time scale to approach and eventually become longer than the SAP time scale near the *T*_g_ range (∼330 to 340 K). This dynamic crossover is reflected in the SCL transformation kinetics, whose apparent activation energy drastically increases with decreasing temperature, reaching ∼530 kJ mol^–1^ in the sub-*T*_g_ temperature range.

## Conclusions

The present work provides an extensive characterization of the transformation kinetics of the poly­(l-lactide) amorphous phase through calorimetric and infrared spectroscopy techniques. Besides standard calorimetry, FSC has been adopted to suppress PLLA cold crystallization and α’ to α crystal reorganization on heating. In this way, a subtle endothermic event in the supercooled liquid (SCL), originating from the so-called SCL transformation, is unveiled. It begins once the glass equilibrates by the main α-relaxation and ends when PLLA nucleates. The annealing kinetics of this relaxation were followed by enthalpic changes of the liquid as well as glass transition temperature variations over the annealing time. The SCL transformation exhibits milder activation energy compared to the α-relaxation, decreasing from 530 kJ mol^–1^ close to *T*_g_ to 130 kJ mol^–1^ at higher temperatures before disappearing above 383 K. Notably, PLLA nucleation kinetics seem to be enslaved to the SCL transformation, occurring only at the end of the liquid relaxation. Time-Resolved Infrared Spectroscopy of PLLA during isothermal annealing highlighted a conformational rearrangement of the disordered phase of PLLA prior to cold crystallization, enriching energy-favorable *gt* conformers at the expense of the high-energy *gg* form. The study of annealing kinetics into a wide temperature interval allowed to unequivocally associate the enthalpic loss of the SCL transformation observed by FSC measurements with the conformational rearrangement of *gg* conformers observed by FTIR. Finally, these results show a hierarchical relaxation sequence, comprising the α-relaxation as the fastest molecular mechanism, followed by the SCL transformation, nucleation, and, ultimately, crystal growth.

## Supplementary Material



## Notes

Detailed parameters for step-response protocol and time-resolved FTIR isothermal annealing; DSC and FSC thermograms for isothermal annealing of PLLA; enthalpy landscape of amorphous PLLA, calculated from FSC thermograms; and glass transition displacement for PLLA with isothermal annealing (PDF)
