# Supplementary material for: Multistep Relaxation Pathway from Glass to Crystal Development in Poly(l‑lactide)
Source: Macromolecules. 2025 Dec 12;58(24):12966–80. doi: 10.1021/acs.macromol.5c02464 (PMC12752705; doi:10.1021/acs.macromol.5c02464)
Supplement: Supplementary file 1 [file ma5c02464_si_001.pdf]

SUPPORTING INFORMATION

Multi-step relaxation pathway from glass to crystal development in poly(L-lactide)

Lorenzo Augusto Rocchi[1*], Elisa Sturabotti[1], Andrea Martinelli[1], Daniele Cangialosi[2,3], and Valerio Di Lisio[2,3*]

1. *Department of Chemistry, Sapienza University of Rome, P.le Aldo Moro 5, 00185 Rome ,Italy*
2. *Donostia International Physics Center, P. Manuel Lardizabal 4, 20018 Donostia/San Sebastian, Spain*
3. *Material Physics Center, P. Manuel Lardizabal 5, 20018 Donostia/San Sebastian, Spain*

LIST OF CONTENTS

Step-response parameters 2

Time Resolved FT-IR isothermal annealing parameters 2

DSC thermograms for isothermal annealing 3

FSC $c_p – c_p^{ref}$ curves for isothermal annealing 4

Enthalpy mapping of quenched and annealed PLLA 5

Glass transition displacement with annealing 8

REFERENCES 9

Step-response parameters

Table S1 *Step response experimental parameters: q_H heating rate, t_{iso} isotherm duration, t_p stimulation cycle period, and f_o base frequency, and ω_o base angular frequency.*

	q_H (K/s)	t_{iso} (s)	t_p (s)	f_o (Hz)	ω_o (rad/s)
**FSC**	1000	0.1004	0.1024	9.77	61.3
	400	1.6334	1.6384	0.61	3.83
**DSC**	0.25	120	128	0.00781	0.049
	0.03125	960	1024	0.000977	0.0061
	0.0833	7956	8196	0.000122	0.00077

Time Resolved FT-IR isothermal annealing parameters

Table S2 *TR-FTIR experimental parameters: film thickness, T_a annealing temperature, and t_a annealing time.*

Film thickness (μm)	T_a (K)	t_a (h)
6	329.2	162
8	331.5	308
7	334.0	112
5	336.0	64
9	339.0	64
7	341.5	64
10	342.6	39
6	346.8	67
8	349.2	41

DSC thermograms for isothermal annealing

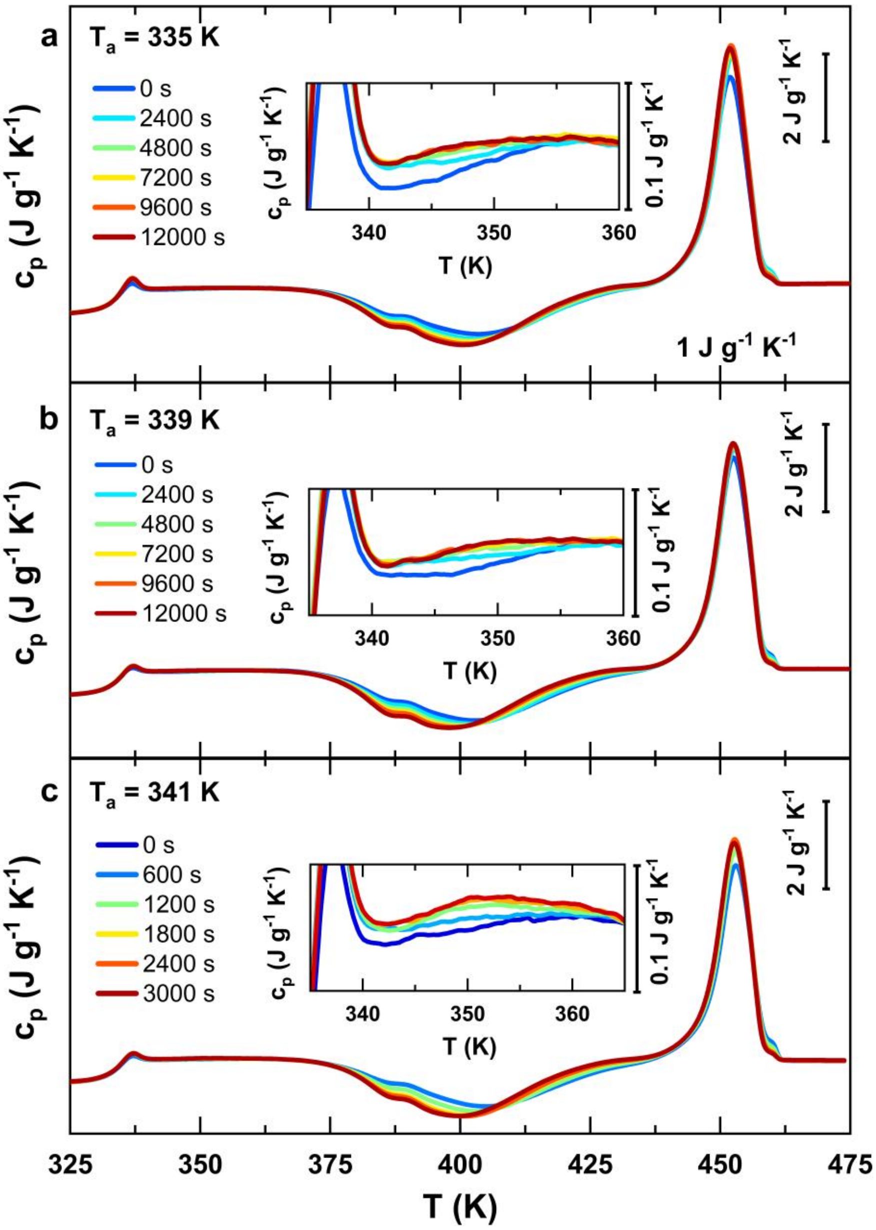

Figure S1. DSC thermograms recorded at a heating rate of 10 K min^{-1} for PLLA annealed at T_a for different times. In the insets the SCL transformation endotherms are magnified.

FSC c_p – c_p^{ref} curves for isothermal annealing

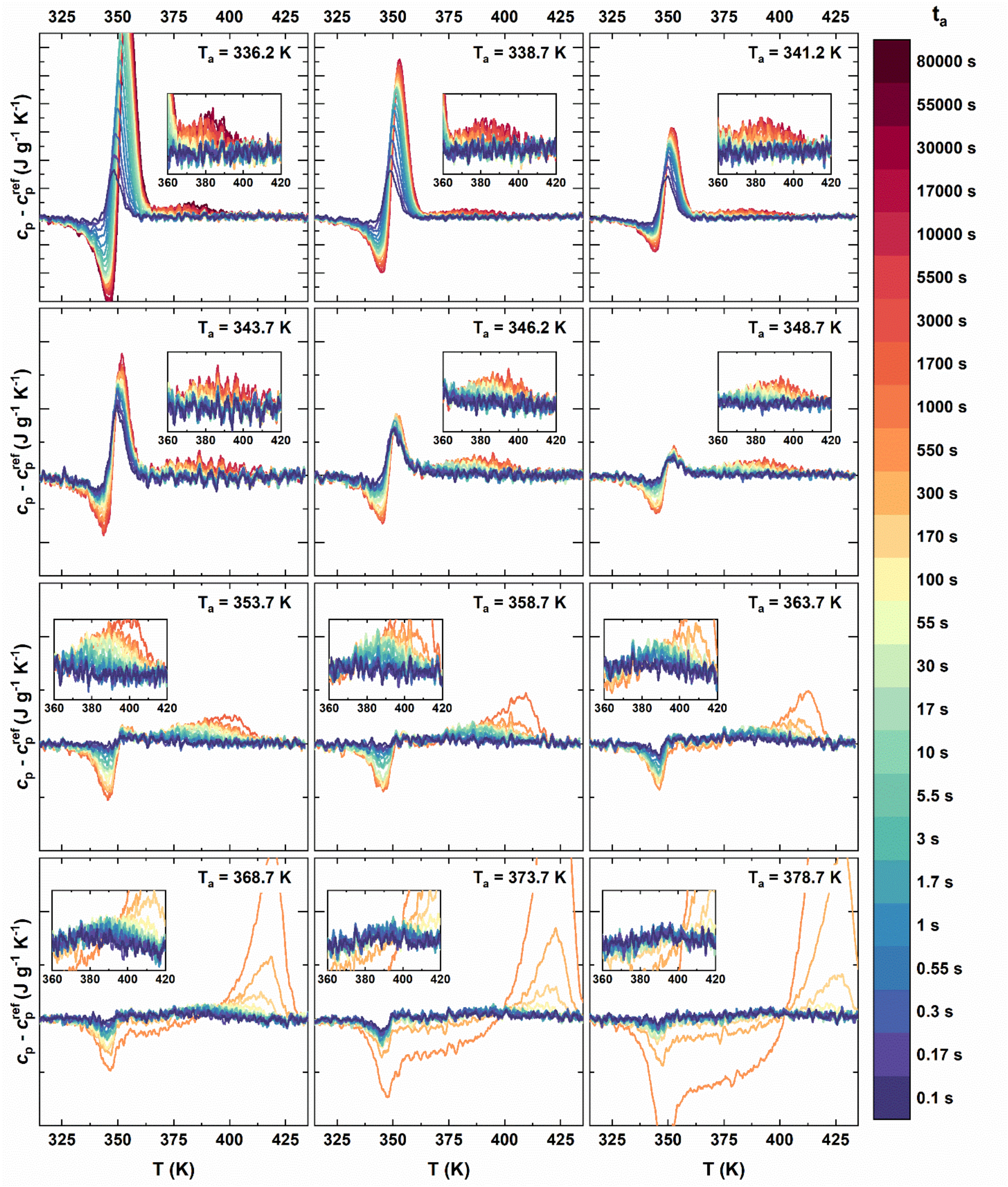

Figure S2. FSC c_p - c_p^{ref} curves recorded at all explored T_a for various annealing time t_a. Insets highlight the SCL transformation endotherm. Major thicks represent a scale of 0.1 J g^{-1} K^{-1}.

Enthalpy mapping of quenched and annealed PLLA

The enthalpy map of PLLA is built from the c_p cooling curve of the quenched sample (c_p^Q), that is, cooled at -1000 K s^{-1}, shown by the black line in Figure S3a. Additionally, the specific heat of the liquid line (c_p^{liq}) is represented in red, and it was determined by the equation taken from Pyda et al.:[1]

$$c_p^{liq}(T)\ [J g^{-1} K^{-1}] = \frac{(120.17+0.076\cdot T)\ [J\ mol^{-1}\ K^{-1}]}{72\ [g\ mol^{-1}]} \qquad \text{Eq. S2}$$

Where 72 g mol^{-1} is the molecular weight of the PLLA repeating unit.

The integration over temperature of c_p^Q and of the c_p^{liq} gives the enthalpy of the PLLA quenching curve, H, as well as the enthalpy of the supercooled liquid line H_{SCL}. These were determined by the equations S3 and S4 and plotted in Figure S3b:

$$H(T) = H_0 + \int_{T_0}^{T} c_p^Q\, dT' \qquad \text{Eq. S3}$$

$$H_{SCL}(T) = H_0' + \int_{T_0}^{T} c_p^{liq}\, dT' \qquad \text{Eq. S4}$$

With $H_0' = H_0 - 42.0\, J\, g^{-1}$ Eq. S5

Where the starting integration temperature T_0 was chosen to be 273 K, H_0 and H'_0 are the absolute enthalpies at 273 K of the quenched PLLA and of the extrapolated liquid, respectively. To achieve the equivalence between H(T) and H_{SCL}(T) above T_g (T > 360 K), H'_0 and H_0 were chosen accordingly (see Eq. S5). This means that the enthalpy of the supercooled liquid resulted to be diminished by 42.0 J g^{-1} at 273 K with respect to the enthalpy of the quenched PLLA.

Finally, the equilibrated enthalpies reached by annealing after glass annealing equilibration ($H_{eq,glass}$) and after the end of the SCL transformation ($H_{eq,SCL}$), were calculated according to equations S6 and S7 and added to Figure S3b:

$$H_{eq,glass}(T_a) = H(T_a) - \Delta H_{eq,rec}(T_a) \qquad \text{Eq. S6}$$

$$H_{eq,SCL}(T_a) = H(T_a) - \Delta H_{eq,rec}(T_a) - \Delta H_{eq,SCL}(T_a) \qquad \text{Eq. S7}$$

where $\Delta H_{eq,rec}$ and $\Delta H_{eq,SCL}$ are the equilibrium enthalpy recoveries at the explored annealing temperatures T_a of the overshoot and of the SCL transformation endotherms, respectively, shown in Figure 3c of the main text. $\Delta H_{eq,rec}$ becomes 0 for annealing above T_g (T_a > 348.7 K).

The procedure described in this paragraph enables to map the enthalpy landscape for amorphous PLLA. The detailed discussion of the enthalpy map is reported in the "Thermodynamic stability of PLLA supercooled liquid state" paragraph of the main text.

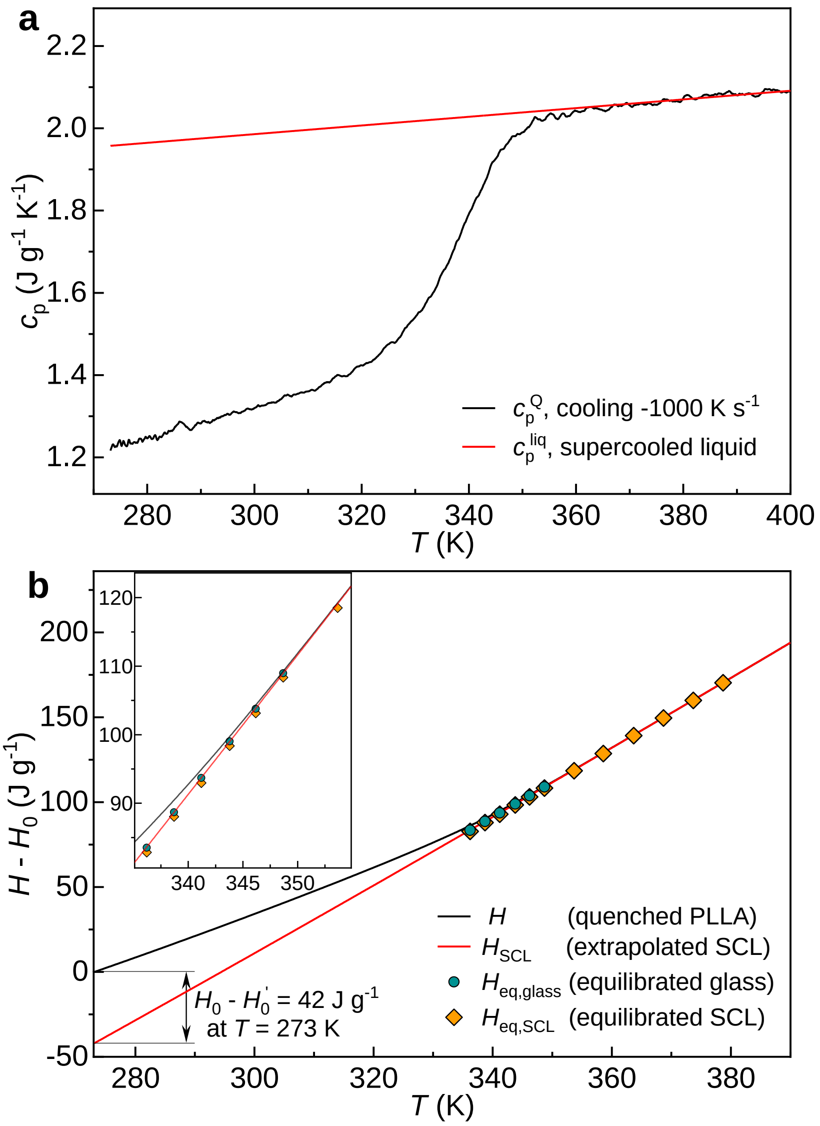

Figure S3. (a) c_p curve (black line) of quenched PLLA, recorded during cooling from 483 K to 273 K at -1000 K s^{-1}. The specific heat curve of the liquid state, taken from Pyda et al,[1] is represented in red. (b) Enthalpy mapping of PLLA quenching (black line) and of the extrapolated supercooled liquid (red line), H_{SCL}, together with equilibrated enthalpies after glass annealing $H_{eq,glass}$, and after the SCL transformation $H_{eq,SCL}$. The inset magnifies the enthalpy map in the glass transition region.

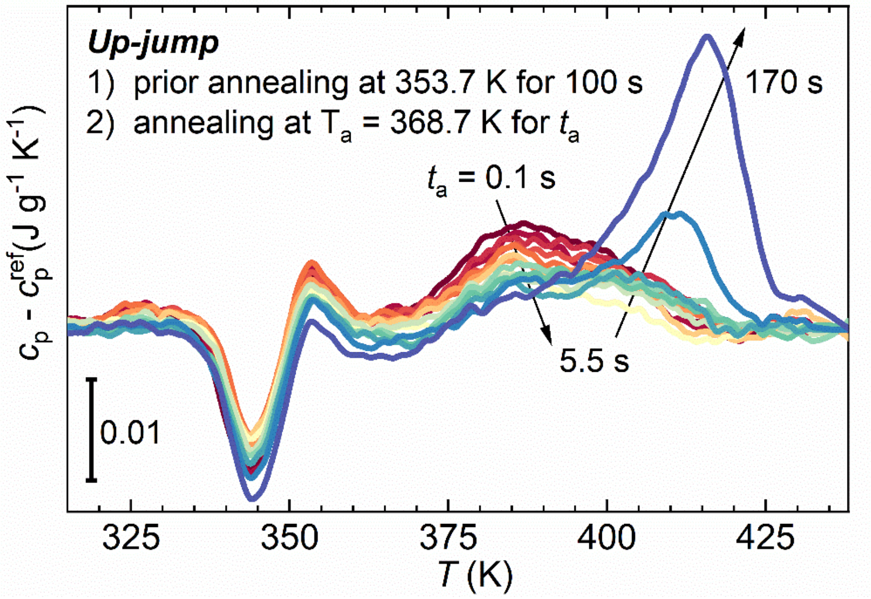

Figure S4. $c_p - c_p^{ref}$ heating curves for the up-jump annealing experiment used to test the asymmetry of approach. After the prior annealing at 353.7 K for 100 s, the characteristic endotherm of the SCL transformation arises, confirming the enthalpy loss of PLLA quenched SCL to reach the relaxed SCL state. The second annealing at 368.7 K for different t_a shows an initial decrease of the SCL endo peak up to 5.5 s, followed by the incipient melting endo peak arising at 415-420 K at the last annealing stages.

Glass transition displacement with annealing

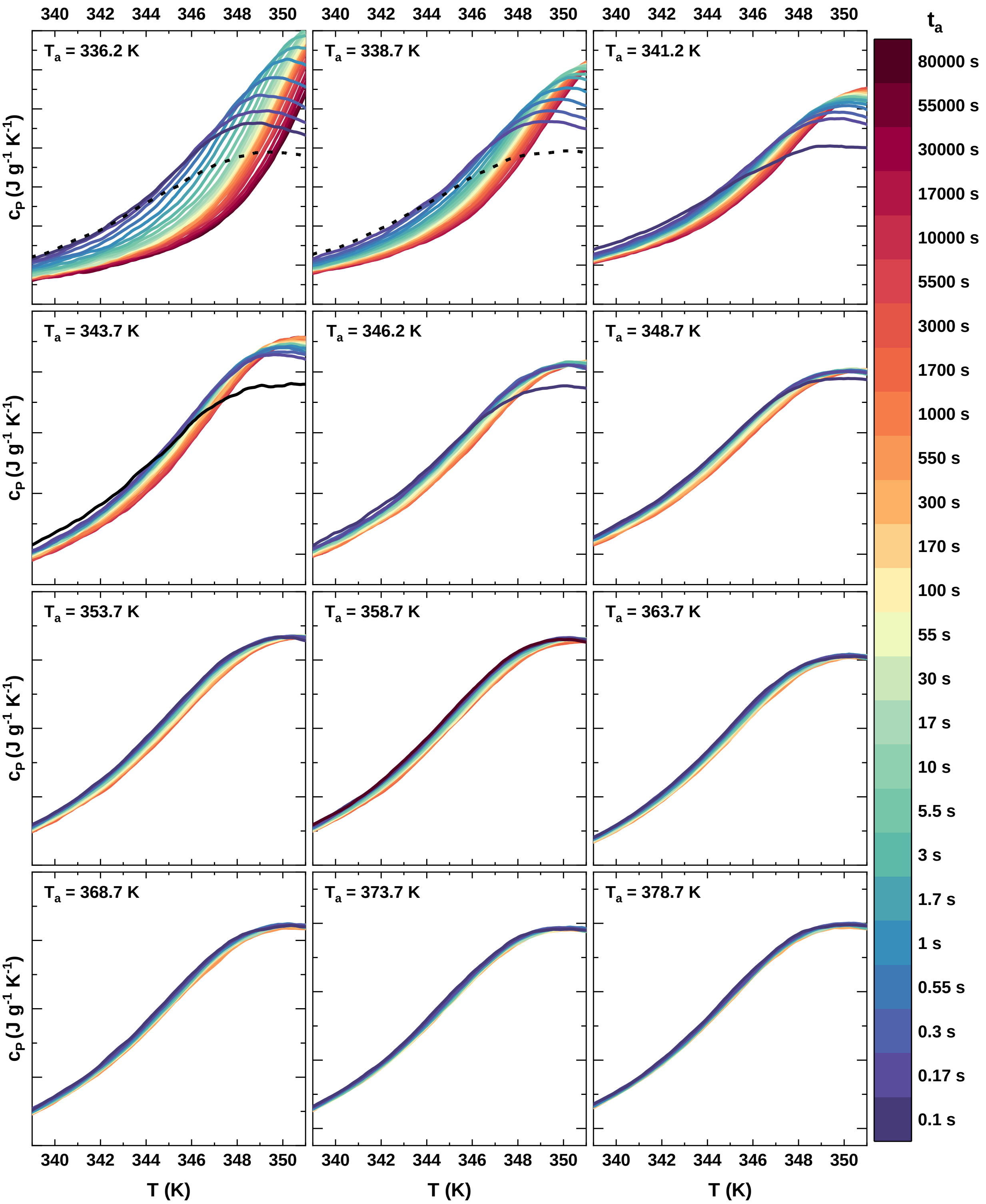

Figure S5. Magnification of the glass transition range of c_p curves after annealing for all the explored T_a and t_a, recorded at 1000 K s^{-1}.

REFERENCES

(1) Pyda, M.; Bopp, R. C.; Wunderlich, B. Heat Capacity of Poly(Lactic Acid). *J Chem Thermodyn* **2004**, *36* (9), 731–742. https://doi.org/10.1016/j.jct.2004.05.003.
